# Identification of activity-induced *Egr3*-dependent genes reveals genes associated with DNA damage response and schizophrenia

**DOI:** 10.1038/s41398-022-02069-8

**Published:** 2022-08-08

**Authors:** Ketan K. Marballi, Khaled Alganem, Samuel J. Brunwasser, Arhem Barkatullah, Kimberly T. Meyers, Janet M. Campbell, Annika B. Ozols, Robert E. Mccullumsmith, Amelia L. Gallitano

**Affiliations:** 1 Department of Basic Medical Sciences, University of Arizona College of Medicine - Phoenix, Phoenix, AZ 85004 USA; 2 Northeastern University, Toronto, ON M5X 1E2 Canada; 3 Department of Neurosciences, University of Toledo College of Medicine and Life Sciences, Toledo, OH USA; 4 Washington University, Washington University School of Medicine, St. Louis, MO 63110 USA; 5 School of Life Sciences, Arizona State University, Tempe, AZ 85287 USA; 6 Department of Neurobiology, Barrow Neurological Institute, Phoenix, AZ 85013 USA; 7 Neurosciences Institute, ProMedica, Toledo, OH USA

**Keywords:** Molecular neuroscience, Schizophrenia

## Abstract

Bioinformatics and network studies have identified the immediate early gene transcription factor early growth response 3 (EGR3) as a master regulator of genes differentially expressed in the brains of patients with neuropsychiatric illnesses ranging from schizophrenia and bipolar disorder to Alzheimer’s disease. However, few studies have identified and validated *Egr3*-dependent genes in the mammalian brain. We have previously shown that *Egr3* is required for stress-responsive behavior, memory, and hippocampal long-term depression in mice. To identify *Egr3*-dependent genes that may regulate these processes, we conducted an expression microarray on hippocampi from wildtype (WT) and *Egr3−/−* mice following electroconvulsive seizure (ECS), a stimulus that induces maximal expression of immediate early genes including *Egr3*. We identified 69 genes that were differentially expressed between WT and *Egr3−/−* mice one hour following ECS. Bioinformatic analyses showed that many of these are altered in, or associated with, schizophrenia, including *Mef2c* and *Calb2*. Enrichr pathway analysis revealed the GADD45 (growth arrest and DNA-damage-inducible) family (*Gadd45b*, *Gadd45g*) as a leading group of differentially expressed genes. Together with differentially expressed genes in the AP-1 transcription factor family genes (*Fos*, *Fosb*), and the centromere organization protein *Cenpa*, these results revealed that *Egr3* is required for activity-dependent expression of genes involved in the DNA damage response. Our findings show that *EGR3* is critical for the expression of genes that are mis-expressed in schizophrenia and reveal a novel requirement for EGR3 in the expression of genes involved in activity-induced DNA damage response.

## Introduction

Major advances in genomics over the past decade have led to identification of hundreds of genes associated with risk for neuropsychiatric illnesses, including schizophrenia, bipolar disorder, depression, and Alzheimer’s dementia. Many of these risk genes are shared across these disorders [1], each of which is characterized by cognitive dysfunction. With the discovery of such a vast number of putative illness-influencing genes, the challenge becomes how to identify the functional relationships that provide insights into the mechanisms underlying neuropsychiatric and neurodegenerative illnesses.

A promising approach has been to define functional networks of genes that are differentially regulated in individuals affected by these illnesses, compared with controls, and then to identify the “Master Regulatory Genes” that best account for these differences in gene expression. The immediate early gene transcription factor early growth response 3 (EGR3) has emerged as such a master regulator of differentially expressed genes (DEGs) in multiple neuropsychiatric disorders including schizophrenia [2], bipolar disorder [3], and most recently Alzheimer’s dementia [4]. However, despite these regulatory relationships identified using bioinformatic approaches, few genes regulated by EGR3 have been validated in the brain in vivo.

One of the first EGR3 downstream target genes to be identified in the brain is activity-regulated cytoskeleton-associated protein (*Arc*) [5]. ARC has been implicated in schizophrenia by studies of rare variants, *de novo* mutations, and single nucleotide polymorphism associations [6–9]. EGR3 is also reported to upregulate glutamic acid decarboxylase A4 (GABRA4) in response to seizure [10, 11]. Neuropathologic studies have identified dysfunction in the GABAergic system in schizophrenia and GABRA4 is also an autism susceptibility gene [12].

Our prior work has identified deficits in the function of ionotropic glutamate receptors in *Egr3−/−* mice, specifically those containing the NMDA2B (GRIN2B) subunit [13]. This indicates that *Egr3* is required for function of a receptor at the center of one of the leading models of schizophrenia pathogenesis, the NMDA (GRIN) receptor hypofunction model of schizophrenia [14]. In this and other studies we found that *Egr3−/−* mice have deficits in stress-responsive behavior, memory, and hippocampal long-term depression, further supporting the importance of *Egr3* in behavioral and electrophysiologic processes implicated in neuropsychiatric disorders and cognitive processes [13, 15].

Based on these findings, we hypothesized that EGR3 is a critical transcriptional regulator in a biological pathway essential for memory, synaptic plasticity, and risk for schizophrenia [16]. As an immediate early gene, EGR3 expression is induced in response to neuronal activity in a manner dependent upon GRIN receptor function and calcium signaling, processes implicated in schizophrenia [17]. EGR3 interacts in regulatory feedback loops with other EGR-family genes in the immune system, including *EGR1*, *EGR2*, *EGR4* and *NAB2*, each of which maps to GWAS loci for schizophrenia [18–21]. Dysfunction in these genes leads to abnormalities in processes that are disrupted in schizophrenia, including memory, synaptic plasticity, immune function, growth factor-mediated processes, myelination, and vascularization [13, 16, 22–29]. Based on the central role of EGR3 in these critical processes, we hypothesized that genes regulated downstream of EGR3 will contribute to risk for schizophrenia and other neuropsychiatric disorders that are characterized by abnormalities in cognition, memory, and synaptic function.

To test this hypothesis, we sought to characterize the complement of genes that require EGR3 in the hippocampus, a critical region for memory formation. We used electroconvulsive seizure (ECS) to maximally activate immediate early gene expression in the hippocampus of *Egr3−/−* and wildtype (WT) mice and conducted an expression microarray to identify genes differentially expressed between the genotypes one hour and two hours following the stimulus, compared to baseline unstimulated conditions. Here, we show that over 69 genes are differentially expressed in the hippocampi of *Egr3−/−* mice compared to WT controls. Several of these *Egr3*-dependent genes map to schizophrenia GWAS loci, and are abnormally expressed in the brains of patients with schizophrenia, bipolar disorder, depression, and Alzheimer’s dementia, supporting findings of studies indicating that EGR3 may be a master regulator of pathophysiological changes in numerous neuropsychiatric disorders [1–4].

## Materials and methods

### Mice

Previously generated *Egr3−/−* mice [30] were backcrossed to C57BL/6 mice for greater than 20 generations. All studies were carried out on homozygous adult progeny (*Egr3−/−* and wildtype (WT)) resulting from heterozygote matings and were assigned as “matched pairs” at the time of weaning. Matched pairs were subjected to identical conditions for all studies. The microarray studies, and quantitative RT-PCR validation studies, were performed on a cohort of male mice ages 6–12 months (*n* = 4 per group). Replication studies were performed on a cohort of female mice, ages 12–15 months (*n* = 4–5 per group). Animals were housed on a 12-hour light/dark cycle with *ad libitum* access to food and water. All studies were performed in accordance with the University of Arizona Institutional Animal Care and Use Committee (IACUC) guidelines under an approved IACUC protocol.

### Electroconvulsive seizure and tissue collection

Electroconvulsive stimulation was delivered to mice via corneal electrodes 5 min following application of 0.5% proparacaine hydrochloride ophthalmic solution (Akorn, Inc., Lake Forest, IL, United States). The cohort of mice used for the microarray study underwent ECS without general anesthesia. The replication cohort of mice underwent ECS following general anesthesia. Isoflurane anesthesia (VetOne, Boise, ID, United States) was administered in an enclosed chamber at a flow rate of 0.5 mL/min in oxygen. Animals were removed from the chamber after 2 min of complete anesthetization, transferred to room air to recover to a level of light anesthesia, and then administered electrical stimulation via orbital electrodes. Stimulation was administered with a pulse frequency of 260 Hz, pulse width of 0.3 ms, duration of 100 ms and current of 80 mA using an Ugo Basile instrument (Varese, Italy). Mice were observed to undergo tonic-clonic seizure and were placed in their home cage to recover for either one hour or two hours prior to sacrifice. Animals that did not display full tonic-clonic seizure were excluded. Control animals remained in their home cages undisturbed until the time of sacrifice.

### Tissue collection and RNA isolation

Animals were sacrificed using isoflurane overdose, followed by decapitation. The brains were removed, rinsed in ice-cold phosphate buffered saline (PBS), and hemisected along the central sulcus into right and left hemispheres for further studies to quantify mRNA expression. Whole hippocampi were rapidly dissected and immediately placed in RNAlater (Ambion, Waltham, MA, United States). Tissue was transferred to 1.5-mL Eppendorf tubes, frozen on dry ice and then stored at −80 °C. For the microarray and follow-up qRT-PCR studies, RNA was isolated using TRIzol reagent (Life Technologies, Carlsbad, CA, United States) per the manufacturer’s protocol. RNA was resuspended in RNAse-free water and quantitated by spectrophotometry. RNA quality and concentration were determined using an Agilent Bioanalyzer 2100 prior to microarray analysis and reverse transcription for qRT-PCR. An aliquot of the RNA samples was sent to the Microarray Resource Center, Yale/NIH Neuroscience Microarray Center (New Haven, CT, United States) for analysis using an Illumina Mouse WG6 v3.0 expression beadchip microarray. For the replication cohort, RNA isolation was performed using TRI reagent (Sigma-Aldrich, St. Louis, MO, United States) and MagMaxTM Total RNA isolation kit (Ambion, Waltham, MA, United States) according to the manufacturer’s protocol, and quantified using the NanoDrop ND-1000 spectrophotometer (Thermo Scientific, Waltham, MA, United States).

### Microarray procedure and analysis

Gene expression analysis was performed using Illumina Mouse WG6 v3.0 expression beadchip microarray and analyzed using two independent microarray analysis methods. Data analysis and quality control was initially performed using GenePattern [31], with normalization using the cubic spline method under the setting False Discover Rate (FDR) < 0.05, to determine significantly DEGs between the WT and *Egr3−/−* groups 1 h and 2 h following ECS. A parallel analysis was performed using the Illumina Genome studio 2010 software to identify DEGs using the following settings: background subtraction, quantile normalization, *p* < 0.05, Illumina custom algorithm. The Illumina custom algorithm uses “Diff scores” to account for reproducibility of results, by *P* value and the magnitude of gene expression difference represented by signal intensity between the reference and control groups [32], where Diff score = 10 X (*Egr3−/−* ECS signal intensity gene A - WT ECS signal intensity gene A) x log_10_ *P* value, where a *P* value > 0.05, would correspond to a diff score of >13 and <−13. To minimize the risk of false positives, the complement of DEGs that was pursued for further analysis was limited to genes that were identified using both methods. Finally, a list of common DEGs between both programs was generated. From this common DEG list, genes that showed a fold change difference of 1.5-fold or higher between the two groups and a *p* value of <0.05, were used for all subsequent analyses. Supplementary Tables S1–S3 show the results of both analyses, as well as the final list of DEGs common to both methods, for each timepoint: baseline, 1 h after ECS, and 2 h after ECS.

### Bioinformatics

Gene expression data are visualized as heatmaps with unsupervised hierarchical clustering, and the values are scaled by row/gene to highlight the differences of gene expression patterns across the samples. To explore clusters of genes that share similar gene expression patterns between the WT and *Egr3−/−* groups across the different time points (baseline, one hour, and two hours after ECS), k-means clustering was performed. Using a range of 1–10, the optimal k was determined as 4, using the elbow method, by calculating the total within sum of square for each k. For the pathway analyses, the Enrichr web tool was used to perform multiple gene set enrichment analyses across different libraries [33]. The gene set libraries used in the pathway analysis are Gene Ontology (GO) Biological Process 2021, GO Molecular Function 2021, BioPlanet 2019, WikiPathways 2019 Mouse, and KEGG 2019 Mouse. The Enrichr pathway analysis bar graphs are sorted by *p* value.

For the schizophrenia lookup studies, the Kaleidoscope web tool was used to extract relevant schizophrenia datasets (https://kalganem.shinyapps.io/Kaleidoscope/). The gene set enrichment analysis was performed by extracting significant genes from each schizophrenia dataset (FDR < 0.05) and using a hypergeometric test to analyze the overlap between the genes differentially expressed in WT and *Egr3−/−* mice 1 h following ECS and the schizophrenia datasets signatures. The hypergeometric test was performed and visualized utilizing the hypeR package [34].

### qRT-PCR

For qRT-PCR studies, mRNA was reverse transcribed into cDNA, as previously described [35], and used as a template for qRT-PCR using FastStart SYBR Green Master mix (Roche Applied Science, Indianapolis, IN, United States) on a 7500 Fast Real-Time PCR machine (ThermoFisher Scientific, Waltham, MA, United States). Each sample was amplified in triplicate for the gene of interest and the housekeeping gene phosphoglycerate kinase 1 (*Pgk1*). *Pgk1* was selected as a housekeeping gene as it showed no significant changes in gene expression across experimental groups in the microarray data. This was validated by qRT-PCR across both male and female cohorts. Gene expression was normalized to *Pgk1* using the 2^−ΔCT^method [36] for each gene of interest to allow comparisons of expression levels between genotypes and across timepoints. Fold-change in gene expression induced by ECS was calculated by dividing post-ECS values by baseline for each genotype independently. Investigators were blinded to genotype and treatment for all qRT-PCR studies.

### Statistical analysis

Sample sizes for the microarray were estimated based on prior studies showing high level induction of gene expression following electrical stimulation [17]. Power analyses performed on the microarray results were used to determine sample sizes to ensure adequate power to detect a large effect size for the qRT-PCR validation and replication studies. Details of microarray and pathway analysis statistics are described in the respective sections. For analysis of qRT-PCR data, we utilized a two-way analysis of variance (ANOVA) followed by Tukey’s *post hoc* test using GraphPad Prism (San Diego, CA, United States) with a significance threshold of *p* < 0.05. Data were plotted as means ± standard error of the mean (SEM). Data were examined graphically within each group, and no strong deviation from normality was observed.

## Results

To identify candidate target genes of EGR3, we conducted an expression microarray of genes differentially expressed in the hippocampus of *Egr3−/−* mice compared with WT controls. Because EGR3 is an activity dependent transcription factor, we used electroconvulsive stimulation to induce a seizure, which maximally actives expression of immediate early genes in the brain [17]. Hippocampal RNA was isolated from *Egr3−/−* and WT mice under three conditions: baseline (no ECS), one hour after ECS, and two hours after ECS. This allowed us to identify genes that require *Egr3* under basal conditions as well as following neuronal activity.

DEGs were determined for each of the three conditions. This included 15 genes at time 0 (hereafter termed “No ECS” or “baseline”), 69 genes one hour after ECS, and 34 genes two hours after ECS. There were a total of 82 DEGs across all three time points (as some genes were differentially expressed at more than one time point). The complete lists of significantly DEGs for each condition are shown in Supplementary Tables S1–S3.

**Fig. 1:**
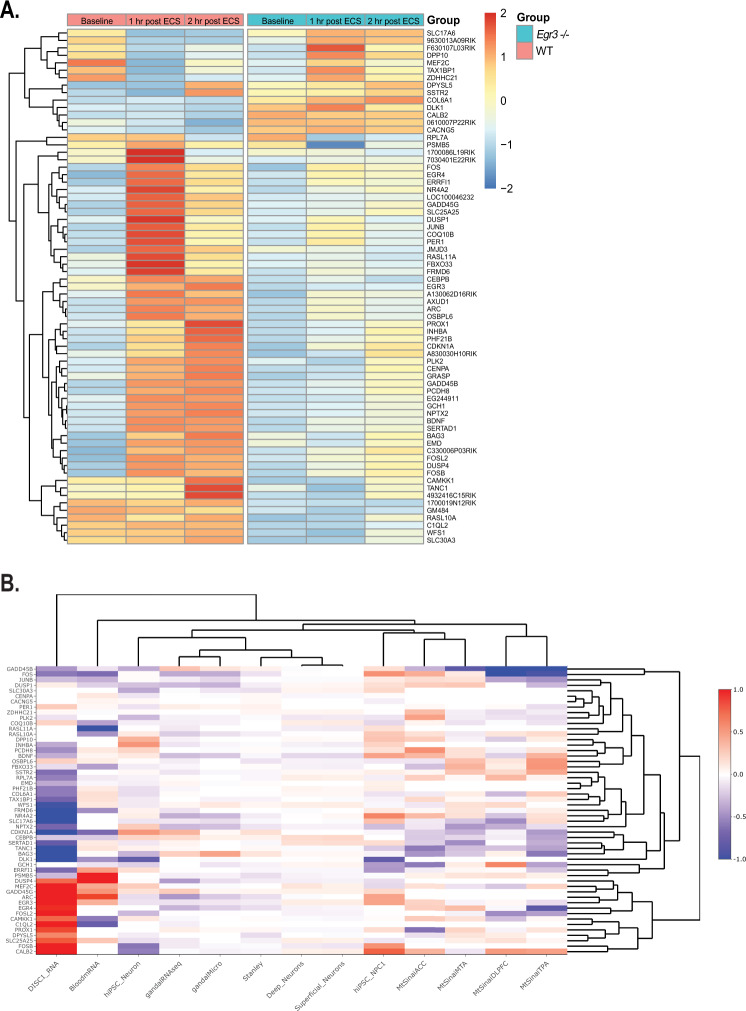
EGR3-dependent genes show altered expression in schizophrenia studies. **A** Expression heatmap of genes differentially expressed in WT versus *Egr3−/−* mice following ECS. Expression microarray revealed 69 genes that were differentially expressed in the hippocampus of *Egr3−/−* mice compared to WT one hour following ECS. The average expression level for each of these genes is shown along a horizontal row in WT (left) and *Egr3−/−* mice (right) at baseline, and 1 h and 2 h following ECS. (*n* = 4 animals per condition). **B** Thirteen published gene expression studies in schizophrenia were queried for the 69 genes that are differentially expressed in *Egr3−/−* compared with WT mouse hippocampus 1 h following ECS. The heatmap shows normalized log2 fold change values (schizophrenia vs. control) from the 13 published study datasets (vertical columns) for each of the 69 EGR3-dependent genes (horizontal rows).

Figure 1A shows a heatmap of the 69 genes that are differentially expressed between *Egr3−/−* and WT mice one hour following ECS, the timepoint with the maximum number of DEGs. Of these, 55 genes were downregulated in *Egr3−/−* mice, while 14 genes were upregulated in *Egr3−/−* mice, compared to WT mice. Table 1 lists the relative expression levels for each DEG identified at 1 h. after ECS. The relative expression level of these 69 DEGs at each time point for each genotype is provided in Supplementary Table S4.

**Table 1:** Genes differentially expressed in the hippocampus of WT versus *Egr3−/−* mice following ECS.

Gene	Protein	Diff score	Fold change (KO ECS/ WT ECS)
*Fosb*	Protein fosB	−335.914	−10.69
*EG244911*	Uncharacterized protein	−335.914	−9.94
*A830030H10Rik*	Riken clone	−335.914	−6.16
*LOC100046232*	Uncharacterized protein	−335.914	−4.71
*Cenpa*	Histone H3-like centromeric protein A	−309.464	−18.28
*Gm484*	Netrin-5	−266.84	−6.79
*Plk2*	Serine/threonine-protein kinase PLK2	−252.231	−2.65
*Fbxo33*	F-box only protein 33	−232.822	−2.58
*Grasp*	GRIP1-associated protein 1	−230.099	−3.38
*Pcdh8*	Protocadherin-8	−202.238	−5.44
*Slc30a3*	Zinc transporter 3	−199.839	−4.73
*Frmd6*	FERM domain-containing protein 6	−185.239	−2.29
*Rasl10a*	Ras-like protein family member 10A	−170.502	−2.71
*Egr3*	Early growth response protein 3	−167.957	−6.89
*Arc*	Activity-regulated cytoskeleton-associated protein	−165.78	−3.99
*Sertad1*	SERTA domain-containing protein 1	−165.07	−3.96
*Phf21b*	PHD finger protein 21B	−154.007	−2.49
*Nr4a2*	Nuclear receptor subfamily 4 group A member 2	−138.367	−2.98
*Camkk1*	Calcium/calmodulin-dependent protein kinase kinase 1	−138.367	−2.03
*C1ql2*	Complement C1q-like protein 2	−133.754	−6.53
*A130062D16Rik*	Riken clone	−130.955	−2.05
*Rpl7a*	60S ribosomal protein L7a	−123.122	−2.41
*Dusp4*	Dual specificity protein phosphatase 4	−118.804	−2.36
*Gadd45g*	Growth arrest and DNA damage-inducible protein GADD45 gamma	−108.553	−2.15
*Bag3*	BAG family molecular chaperone regulator 3	−105.063	−1.85
*Per1*	Period circadian protein homolog 1	−100.752	−1.89
*Rasl11a*	Ras-like protein family member 11A	−90.513	−3.12
*Fos*	Proto-oncogene c-Fos	−80.709	−2.4
*Egr4*	Early growth response protein 4	−79.594	−2.49
*1700019N12Rik*	Riken clone	−70.678	−2.28
*Slc25a25*	Calcium-binding mitochondrial carrier protein SCaMC-2	−65.608	−2.06
*Nptx2*	Neuronal pentraxin-2	−59.585	−8.65
*Coq10b*	Coenzyme Q-binding protein COQ10 homolog B, mitochondrial	−56.113	−1.92
*Fosl2*	Fos-related antigen 2	−53.18	−2.397
*4932416C15Rik*	Riken clone	−44.145	−2.036
*Tanc1*	Protein TANC1	−44.079	−1.66
*1700086L19Rik*	Riken clone	−43.796	−2.69
*Junb*	Transcription factor jun-B	−43.317	−2.44
*Errfi1*	ERBB receptor feedback inhibitor 1	−42.806	−1.97
*Osbpl6*	Oxysterol-binding protein-related protein 6	−40.494	−1.85
*Dusp1*	Dual specificity protein phosphatase 1	−40.162	−2.04
*Bdnf*	Brain-derived neurotrophic factor	−39.862	−3.25
*Wfs1*	Wolframin	−36.559	−1.9
*Prox1*	Prospero homeobox protein 1	−27.356	−1.63
*Jmjd3*	Lysine-specific demethylase 6B	−26.438	−1.96
*C330006P03Rik*	Riken clone	−26.286	−3.47
*Emd*	Emerin	−22.345	−2.19
*Psmb5*	Proteasome subunit beta type-5	−21.268	−2.46
*Gadd45b*	Growth arrest and DNA damage-inducible protein GADD45 beta	−20.227	−4.02
*Gch1*	GTP cyclohydrolase 1	−19.952	−4.46
*Inhba*	Inhibin beta A chain	−19.294	−4.78
*Cdkn1a*	Cyclin-dependent kinase inhibitor 1	−17.694	−1.89
*Cebpb*	CCAAT/enhancer-binding protein beta	−16.31	−2.54
*7030401E22Rik*	Riken clone	−15.74	−2.5
*Axud1*	Cysteine/serine-rich nuclear protein 1	−14.246	−6.28
*Mef2c*	Myocyte-specific enhancer factor 2C	13.14	1.71
*9630013A09Rik*	Riken clone	13.99	1.63
*Zdhhc21*	Probable palmitoyltransferase ZDHHC21	14.253	1.69
*Tax1bp1*	Tax1-binding protein 1 homolog	15.018	1.6
*Dpp10*	Inactive dipeptidyl peptidase 10	15.407	1.78
*Dlk1*	Protein delta homolog 1	17.775	3.71
*Slc17a6*	Vesicular glutamate transporter 2	17.957	1.86
*Calb2*	Calretinin	23.033	1.96
*Col6a1*	Collagen alpha-1(VI) chain	23.071	2.17
*Sstr2*	Somatostatin receptor type 2	24.086	2.56
*0610007P22Rik*	Riken clone	26.781	1.75
*F630107L03Rik*	Riken clone	29.322	1.77
*Cacng5*	Voltage-dependent calcium channel gamma-5 subunit	43.34	2.25
*Dpysl5*	Dihydropyrimidinase-related protein 5	52.13	4.12

69 genes showed a fold change difference of ≥1.5 fold between the two groups with a *p* value of <0.05.

The use of three timepoints (baseline, and 1 and 2 h after ECS) allowed us to perform cluster analyses of all 82 DEGs. Supplementary Fig. S1 shows four major clusters of DEGs, representing genes that display a similar pattern of gene expression changes in WT versus *Egr3−/−* mice over time. Supplementary Figs. S2–S4 and Supplementary Tables S5–S8 show the annotated pathways for the genes identified in each cluster.

### *Egr3*-dependent genes are altered in schizophrenia studies

Next, we tested the original hypothesis for our study, that EGR3 regulates genes that play a role a role in schizophrenia. To do this we examined the expression levels of each of the 69 genes that were differentially expressed at 1 h following ECS in 13 published schizophrenia studies with publicly available datasets [37–43]. These include studies of genes differentially expressed in postmortem brains, peripheral blood, fibroblasts, and induced pluripotent stem cells, from schizophrenia patients compared with controls (annotated in Supplementary Table S9). Gene regulation patterns for the 69 DEGs were assessed across each schizophrenia study. Of these, 58 human homologs were identified in at least one of the schizophrenia datasets. The log fold change in expression of these genes between schizophrenia subjects and control subjects for each of the 58 genes were used to create a heatmap (Fig. 1B). Table 2 lists the fold-change values and adjusted *p* values (controlled for multiple comparisons) of each gene present in each schizophrenia dataset.

**Table 2:** Differential expression of EGR3-dependent genes in schizophrenia studies.

HGNC_Symbol	hiPSC_Neuron	BloodmRNA	DISC1_RNA	gandalMicro	gandalRNAseq
BAG3	−0.32, *p* = 0.0821	0.02, *p* = 0.886	−1.04, *p* = 0.00265	0.46, *p* = 0	0.25, *p* = 0.00118
CDKN1A	0.53, *p* = 0.12105	−0.66, *p* = 0.0386	−1.13, *p* = 4e–05	0.03, *p* = 0.53025	0.38, *p* = 1e–04
TAX1BP1	−0.09, *p* = 0.14238	0.15, *p* = 0.44	−0.69, *p* = 0.00363	−0.05, *p* = 0.00642	0, *p* = 0.80434
DLK1	−0.82, *p* = 0.15185	−0.48, *p* = 0.0211	−2.95, *p* = 0	−0.02, *p* = 0.32619	NA
FOSB	−0.6, *p* = 0.15972	NA	1.61, *p* = 0.05003	−0.03, *p* = 0.50369	−0.09, *p* = 0.30397
FOSL2	−0.3, *p* = 0.16524	NA	1.74, *p* = 0.00028	0.01, *p* = 0.86692	0.01, *p* = 0.89933
PLK2	−0.3, *p* = 0.17343	NA	−0.12, *p* = 0.54524	−0.05, *p* = 0.13323	−0.06, *p* = 0.05341
DUSP1	−0.26, *p* = 0.2098	−0.2, *p* = 0.182	0.09, *p* = 0.76562	−0.33, *p* = 0	−0.3, *p* = 0.05406
CALB2	−0.56, *p* = 0.22733	NA	1.07, *p* = 0.02438	−0.06, *p* = 0.09847	−0.02, *p* = 0.65189
SLC25A25	−0.14, *p* = 0.23559	0.29, *p* = 0.314	0.82, *p* = 0.01491	NA	−0.09, *p* = 0.03948
GADD45B	−0.36, *p* = 0.2407	−0.12, *p* = 0.563	−0.45, *p* = 0.53432	0.13, *p* = 0.03043	0.31, *p* = 0.00108
COL6A1	−0.15, *p* = 0.2678	0.17, *p* = 0.408	−0.57, *p* = 0.02524	0, *p* = 0.89158	0.02, *p* = 0.59097
FBXO33	−0.08, *p* = 0.30964	−0.42, *p* = 0.17	−0.15, *p* = 0.59998	NA	−0.04, *p* = 0.18858
SLC30A3	−0.38, *p* = 0.31862	NA	NA	−0.11, *p* = 0	−0.02, *p* = 0.59594
MEF2C	0.26, *p* = 0.36873	0.44, *p* = 0.128	3.17, *p* = 1e–05	−0.02, *p* = 0.35728	−0.02, *p* = 0.42203
INHBA	0.54, *p* = 0.37038	NA	−0.37, *p* = 0.5783	−0.04, *p* = 0.0793	−0.11, *p* = 0.58146
DPP10	0.4, *p* = 0.39287	NA	NA	NA	−0.05, *p* = 0.0313
WFS1	−0.09, *p* = 0.39648	0.12, *p* = 0.35	−0.97, *p* = 0.0011	0.12, *p* = 1e–05	0.05, *p* = 0.3688
CEBPB	0.22, *p* = 0.4197	−0.22, *p* = 0.288	−0.23, *p* = 0.66459	0.08, *p* = 0.00744	0.19, *p* = 6e–05
PHF21B	−0.14, *p* = 0.4388	0.11, *p* = 0.424	−0.57, *p* = 0.02009	NA	−0.04, *p* = 0.28097
CAMKK1	0.12, *p* = 0.45333	−0.58, *p* = 0.0259	0.75, *p* = 0.0098	NA	−0.03, *p* = 0.23652
PCDH8	0.22, *p* = 0.49638	0.12, *p* = 0.414	−0.49, *p* = 0.1538	−0.02, *p* = 0.62934	0, *p* = 0.96045
EMD	−0.07, *p* = 0.49802	−0.04, *p* = 0.776	−0.37, *p* = 0.06911	−0.02, *p* = 0.10924	0.05, *p* = 0.45742
GCH1	−0.2, *p* = 0.51753	−0.12, *p* = 0.529	NA	−0.11, *p* = 0.00099	−0.01, *p* = 0.79597
NPTX2	0.27, *p* = 0.52891	NA	−0.68, *p* = 0.08796	−0.27, *p* = 0	−0.3, *p* = 1e–05
DPYSL5	−0.08, *p* = 0.54062	NA	0.61, *p* = 0.04561	NA	0, *p* = 0.98678
PER1	−0.12, *p* = 0.55705	0.04, *p* = 0.816	0.26, *p* = 0.43307	0.06, *p* = 0.01761	0.09, *p* = 0.05364
COQ10B	−0.07, *p* = 0.56159	−0.41, *p* = 0.286	0.23, *p* = 0.55183	−0.05, *p* = 0.02826	−0.07, *p* = 0.03374
RPL7A	−0.05, *p* = 0.61042	−0.09, *p* = 0.288	−0.55, *p* = 0.03022	NA	0.02, *p* = 0.34245
SERTAD1	−0.1, *p* = 0.62188	0.11, *p* = 0.605	−0.8, *p* = 0.09215	NA	0.06, *p* = 0.23966
FRMD6	0.07, *p* = 0.65809	−0.49, *p* = 0.129	−1.66, *p* = 0.00086	NA	−0.12, *p* = 0.00013
BDNF	0.12, *p* = 0.66419	0.06, *p* = 0.695	−0.35, *p* = 0.68472	−0.24, *p* = 0	−0.28, *p* = 0.12699
GADD45G	0.15, *p* = 0.66505	0.54, *p* = 0.109	1.21, *p* = 0.00035	0.05, *p* = 0.11504	0.27, *p* = 0.00038
CENPA	−0.11, *p* = 0.67123	0.12, *p* = 0.378	NA	−0.02, *p* = 0.28862	NA
ARC	−0.15, *p* = 0.67385	0.9, *p* = 0.0139	1.34, *p* = 0.05719	−0.23, *p* = 2e–05	−0.45, *p* = 0.01904
ERRFI1	0.17, *p* = 0.68918	0.49, *p* = 0.0108	−0.66, *p* = 0.04065	NA	0.01, *p* = 0.67131
CSRNP1	−0.06, *p* = 0.70835	0.08, *p* = 0.728	−0.41, *p* = 0.22397	NA	0.04, *p* = 0.62456
KDM6B	0.04, *p* = 0.72218	−1.03, *p* = 0.0345	0.31, *p* = 0.19838	0.01, *p* = 0.56945	0.01, *p* = 0.81955
EGR3	−0.12, *p* = 0.73785	0.4, *p* = 0.433	1.64, *p* = 0.00449	−0.1, *p* = 0.00189	−0.12, *p* = 0.15247
TANC1	0.07, *p* = 0.75881	0.13, *p* = 0.595	−1.24, *p* = 8e–05	NA	0.03, *p* = 0.32526
RASL10A	0.11, *p* = 0.76405	−0.47, *p* = 0.0296	NA	−0.13, *p* = 0	−0.22, *p* = 0.01732
SLC17A6	−0.11, *p* = 0.80324	NA	−2.72, *p* = 9e–05	−0.15, *p* = 0.00238	−0.1, *p* = 0.02375
TAMALIN	0.05, *p* = 0.81867	−0.01, *p* = 0.935	1.64, *p* = 0.01204	NA	−0.15, *p* = 0.0281
ZDHHC21	0.02, *p* = 0.82655	−0.02, *p* = 0.954	0.03, *p* = 0.93476	NA	0, *p* = 0.90342
NR4A2	0.09, *p* = 0.82964	NA	−1.69, *p* = 0.01638	−0.3, *p* = 0	−0.35, *p* = 0.13475
OSBPL6	0.02, *p* = 0.86875	0.1, *p* = 0.649	0.24, *p* = 0.33185	NA	−0.08, *p* = 0.05614
DUSP4	0.02, *p* = 0.90675	1.76, *p* = 0	0.74, *p* = 0.02288	−0.15, *p* = 0.00037	−0.43, *p* = 0.09233
SSTR2	0.03, *p* = 0.9106	NA	−0.63, *p* = 0.10038	−0.03, *p* = 0.31275	−0.11, *p* = 0.00136
NTN5	−0.02, *p* = 0.92996	NA	−0.39, *p* = 0.70089	NA	NA
CACNG5	0.03, *p* = 0.93279	0.06, *p* = 0.593	NA	0, *p* = 0.86722	NA
JUNB	0.02, *p* = 0.94177	−0.34, *p* = 0.0843	−0.28, *p* = 0.55915	−0.17, *p* = 0.00088	−0.08, *p* = 0.46257
FOS	0.02, *p* = 0.95035	−0.65, *p* = 0.13	−0.59, *p* = 0.03179	−0.36, *p* = 0.00057	−0.37, *p* = 0.10822
PROX1	−0.01, *p* = 0.961	NA	0.88, *p* = 0.0397	0.03, *p* = 0.37899	0.09, *p* = 0.00831
RASL11A	0.01, *p* = 0.96522	−1.17, *p* = 0.00072	NA	NA	−0.09, *p* = 0.05543
PSMB5	0, *p* = 0.97554	1.45, *p* = 2e–05	−0.29, *p* = 0.33954	−0.05, *p* = 0.01003	−0.07, *p* = 0.00177
C1QL2	NA	−0.78, *p* = 0.00734	6.04, *p* = 0	NA	−0.07, *p* = 0.10395
EGR4	NA	NA	0.97, *p* = 0.30657	−0.2, *p* = 0	−0.32, *p* = 0.02189
C2CD4A	NA	NA	NA	NA	NA

HGNC_Symbol	hiPSC_NPC1	MtSinaiACC	MtSinaiDLPFC	MtSinaiMTA	MtSinaiTPA
BAG3	−0.05, *p* = 0.79517	−0.46, *p* = 0.25869	0.08, *p* = 0.90499	−0.24, *p* = 0.42096	−0.59, *p* = 0.15172
CDKN1A	−0.04, *p* = 0.90292	−0.22, *p* = 0.46986	−0.06, *p* = 0.88564	−0.32, *p* = 0.51548	−0.44, *p* = 0.36054
TAX1BP1	−0.05, *p* = 0.4067	0.2, *p* = 0.01278	−0.04, *p* = 0.68386	0.03, *p* = 0.79524	0.1, *p* = 0.12898
DLK1	−0.81, *p* = 0.28279	NA	NA	NA	NA
FOSB	0.56, *p* = 0.22477	NA	NA	NA	NA
FOSL2	0.01, *p* = 0.96237	−0.01, *p* = 0.95062	−0.5, *p* = 0.02372	−0.06, *p* = 0.72718	−0.42, *p* = 0.16922
PLK2	−0.11, *p* = 0.61775	0.48, *p* = 0.03211	−0.12, *p* = 0.59213	−0.08, *p* = 0.61198	−0.14, *p* = 0.43879
DUSP1	0.17, *p* = 0.42677	0.26, *p* = 0.39728	−0.01, *p* = 0.97916	0.28, *p* = 0.35604	−0.31, *p* = 0.25042
CALB2	0.78, *p* = 0.17148	0.38, *p* = 0.18307	0.51, *p* = 0.17976	−0.11, *p* = 0.58751	0.48, *p* = 0.03202
SLC25A25	−0.11, *p* = 0.37888	−0.02, *p* = 0.88637	0.15, *p* = 0.34145	0.09, *p* = 0.39415	0.05, *p* = 0.82565
GADD45B	0.17, *p* = 0.59048	−0.33, *p* = 0.25363	−1.05, *p* = 0.25174	−0.85, *p* = 0.14295	−1.35, *p* = 0.09954
COL6A1	−0.12, *p* = 0.39612	−0.09, *p* = 0.59109	−0.29, *p* = 0.07906	0.1, *p* = 0.44689	0.26, *p* = 0.04108
FBXO33	−0.01, *p* = 0.87047	−0.27, *p* = 0.16728	0.1, *p* = 0.4471	0.39, *p* = 0.00934	0.54, *p* = 0.0103
SLC30A3	0.2, *p* = 0.63178	NA	NA	NA	NA
MEF2C	−0.16, *p* = 0.59546	0.16, *p* = 0.43094	−0.24, *p* = 0.25495	−0.12, *p* = 0.53305	0.22, *p* = 0.24237
INHBA	0.23, *p* = 0.75278	NA	NA	NA	NA
DPP10	0.31, *p* = 0.54713	0.26, *p* = 0.27222	−0.22, *p* = 0.24723	−0.05, *p* = 0.77682	0.1, *p* = 0.57767
WFS1	−0.03, *p* = 0.77454	−0.1, *p* = 0.36303	0.2, *p* = 0.51505	0.15, *p* = 0.21049	0.01, *p* = 0.9372
CEBPB	0, *p* = 0.99652	−0.23, *p* = 0.23533	−0.08, *p* = 0.69098	−0.17, *p* = 0.41913	−0.39, *p* = 0.09009
PHF21B	0.09, *p* = 0.6062	NA	NA	NA	NA
CAMKK1	0.02, *p* = 0.92176	0.27, *p* = 0.18205	−0.27, *p* = 0.03174	0.16, *p* = 0.35829	0.04, *p* = 0.80671
PCDH8	0.15, *p* = 0.63565	0.57, *p* = 0.03882	−0.06, *p* = 0.80466	0.15, *p* = 0.58969	0.14, *p* = 0.59105
EMD	0.08, *p* = 0.44547	NA	NA	NA	NA
GCH1	−0.43, *p* = 0.19325	−0.58, *p* = 0.13237	0.61, *p* = 0.23203	0.1, *p* = 0.70762	−0.41, *p* = 0.21419
NPTX2	−0.28, *p* = 0.51109	−0.1, *p* = 0.67103	−0.35, *p* = 0.2751	−0.45, *p* = 0.2745	−0.15, *p* = 0.7255
DPYSL5	−0.01, *p* = 0.91137	−0.13, *p* = 0.51241	0.1, *p* = 0.71991	0.14, *p* = 0.39232	−0.01, *p* = 0.90619
PER1	0, *p* = 0.99052	0.03, *p* = 0.76349	0.02, *p* = 0.90763	0, *p* = 0.99099	−0.14, *p* = 0.19271
COQ10B	−0.17, *p* = 0.18681	0.06, *p* = 0.55984	−0.02, *p* = 0.78616	−0.12, *p* = 0.2149	−0.12, *p* = 0.25397
RPL7A	−0.07, *p* = 0.52573	0.25, *p* = 0.13596	0.36, *p* = 0.26567	0.04, *p* = 0.7178	0.07, *p* = 0.38373
SERTAD1	0.18, *p* = 0.37682	−0.21, *p* = 0.35465	−0.01, *p* = 0.98325	−0.49, *p* = 0.0898	−0.35, *p* = 0.21307
FRMD6	0.12, *p* = 0.39499	0.04, *p* = 0.82184	−0.03, *p* = 0.87729	0.14, *p* = 0.29411	0.09, *p* = 0.53324
BDNF	0.47, *p* = 0.11501	0.37, *p* = 0.24235	−0.2, *p* = 0.60573	0.16, *p* = 0.66307	0.39, *p* = 0.19732
GADD45G	0.2, *p* = 0.546	NA	NA	NA	NA
CENPA	0.02, *p* = 0.91488	NA	NA	NA	NA
ARC	0.47, *p* = 0.22708	NA	NA	NA	NA
ERRFI1	−0.45, *p* = 0.29955	−0.08, *p* = 0.52989	0.02, *p* = 0.8967	0.04, *p* = 0.49242	−0.24, *p* = 0.11842
CSRNP1	−0.1, *p* = 0.61371	0.06, *p* = 0.77413	−0.33, *p* = 0.26213	−0.3, *p* = 0.11005	−0.4, *p* = 0.17261
KDM6B	−0.01, *p* = 0.95267	0.21, *p* = 0.24157	0.05, *p* = 0.78434	−0.09, *p* = 0.61973	−0.26, *p* = 0.22944
EGR3	0.46, *p* = 0.2355	0.23, *p* = 0.26581	0.03, *p* = 0.88893	−0.05, *p* = 0.79828	−0.15, *p* = 0.56398
TANC1	−0.23, *p* = 0.35367	−0.57, *p* = 0.0152	−0.26, *p* = 0.13805	−0.27, *p* = 0.14701	−0.4, *p* = 0.06464
RASL10A	0.28, *p* = 0.55199	0.16, *p* = 0.44907	0.13, *p* = 0.51148	−0.09, *p* = 0.75454	0.19, *p* = 0.36989
SLC17A6	0.33, *p* = 0.50584	−0.09, *p* = 0.76637	−0.5, *p* = 0.01389	−0.25, *p* = 0.20713	0.19, *p* = 0.43558
TAMALIN	−0.15, *p* = 0.472	0.29, *p* = 0.18464	−0.14, *p* = 0.35768	0.2, *p* = 0.19358	0.2, *p* = 0.29512
ZDHHC21	0.01, *p* = 0.93489	0.34, *p* = 0.01416	−0.15, *p* = 0.38666	−0.07, *p* = 0.68939	−0.08, *p* = 0.58333
NR4A2	0.62, *p* = 0.16673	0.35, *p* = 0.16386	−0.25, *p* = 0.45198	0.15, *p* = 0.5745	0.15, *p* = 0.54504
OSBPL6	−0.1, *p* = 0.42634	−0.11, *p* = 0.46335	0.13, *p* = 0.38738	0.33, *p* = 0.06354	0.52, *p* = 0.00106
DUSP4	−0.15, *p* = 0.45815	NA	NA	NA	NA
SSTR2	0.09, *p* = 0.72974	0.05, *p* = 0.81328	0.2, *p* = 0.34572	0.31, *p* = 0.23386	0.45, *p* = 0.00646
NTN5	−0.1, *p* = 0.75761	NA	NA	NA	NA
CACNG5	−0.07, *p* = 0.86759	NA	NA	NA	NA
JUNB	0.11, *p* = 0.64975	0.18, *p* = 0.48983	−0.4, *p* = 0.50242	0.16, *p* = 0.48992	−0.48, *p* = 0.26895
FOS	0.56, *p* = 0.14087	0.42, *p* = 0.42354	−1.15, *p* = 0.39057	0.11, *p* = 0.80601	−0.94, *p* = 0.22862
PROX1	0.18, *p* = 0.53785	−0.62, *p* = 0.06257	−0.05, *p* = 0.8297	0.22, *p* = 0.38952	0.14, *p* = 0.5746
RASL11A	0.15, *p* = 0.59965	NA	NA	NA	NA
PSMB5	−0.02, *p* = 0.84252	0.08, *p* = 0.48029	0.16, *p* = 0.34105	0.13, *p* = 0.28466	0.08, *p* = 0.44466
C1QL2	NA	NA	NA	NA	NA
EGR4	NA	0.25, *p* = 0.3989	−0.03, *p* = 0.94403	0.34, *p* = 0.17492	−0.85, *p* = 0.23701
C2CD4A	NA	NA	NA	NA	NA

HGNC_Symbol	Stanley	Deep_Neurons	Superficial_Neurons
BAG3	0.13, *p* = 0.18171	0.04, *p* = 0.69947	0.04, *p* = 0.72516
CDKN1A	0.17, *p* = 0.08039	0.01, *p* = 0.95415	0, *p* = 0.98405
TAX1BP1	−0.05, *p* = 0.19246	−0.01, *p* = 0.92427	−0.02, *p* = 0.89588
DLK1	0, *p* = 0.90792	0.01, *p* = 0.95252	0.02, *p* = 0.83855
FOSB	−0.02, *p* = 0.63824	−0.09, *p* = 0.39393	−0.11, *p* = 0.33335
FOSL2	−0.01, *p* = 0.64448	−0.05, *p* = 0.4799	−0.05, *p* = 0.50787
PLK2	−0.02, *p* = 0.80023	−0.1, *p* = 0.37039	−0.09, *p* = 0.41683
DUSP1	−0.07, *p* = 0.24855	0.04, *p* = 0.5955	0.05, *p* = 0.45598
CALB2	−0.04, *p* = 0.53085	0.08, *p* = 0.21986	0.09, *p* = 0.2113
SLC25A25	−0.06, *p* = 0.33908	0.1, *p* = 0.36655	0.08, *p* = 0.44629
GADD45B	0.07, *p* = 0.0797	−0.02, *p* = 0.8695	−0.01, *p* = 0.96015
COL6A1	0.01, *p* = 0.20955	0.04, *p* = 0.64557	0.04, *p* = 0.67095
FBXO33	0.02, *p* = 0.15155	−0.06, *p* = 0.45867	−0.07, *p* = 0.38679
SLC30A3	−0.13, *p* = 0.15692	0.06, *p* = 0.41934	0.07, *p* = 0.39729
MEF2C	0, *p* = 0.96954	0.13, *p* = 0.5087	0.08, *p* = 0.6774
INHBA	−0.02, *p* = 0.45229	−0.06, *p* = 0.43342	−0.08, *p* = 0.3049
DPP10	0, *p* = 0.90157	0.03, *p* = 0.71465	0.02, *p* = 0.78353
WFS1	0.05, *p* = 0.31329	0.09, *p* = 0.17783	0.1, *p* = 0.14681
CEBPB	0.07, *p* = 0.31916	0.19, *p* = 0.0686	0.21, *p* = 0.0487
PHF21B	NA	0.06, *p* = 0.44825	0.04, *p* = 0.56301
CAMKK1	0.02, *p* = 0.81091	−0.02, *p* = 0.7595	−0.03, *p* = 0.64491
PCDH8	0.02, *p* = 0.82012	−0.02, *p* = 0.82115	−0.02, *p* = 0.85006
EMD	0.02, *p* = 0.58101	NA	NA
GCH1	−0.05, *p* = 0.26007	0.05, *p* = 0.49696	0.06, *p* = 0.42438
NPTX2	−0.11, *p* = 0.14812	−0.05, *p* = 0.5487	−0.05, *p* = 0.51358
DPYSL5	−0.15, *p* = 0.02334	−0.01, *p* = 0.92474	−0.02, *p* = 0.82193
PER1	0.07, *p* = 0.13636	NA	NA
COQ10B	−0.01, *p* = 0.69119	0.1, *p* = 0.35391	0.11, *p* = 0.34034
RPL7A	−0.04, *p* = 0.3839	0.09, *p* = 0.33883	0.08, *p* = 0.37785
SERTAD1	−0.03, *p* = 0.47644	0.17, *p* = 0.01527	0.18, *p* = 0.01181
FRMD6	0, *p* = 0.97149	−0.02, *p* = 0.81704	0, *p* = 0.99211
BDNF	−0.03, *p* = 0.31063	−0.16, *p* = 0.15481	−0.16, *p* = 0.15571
GADD45G	0.06, *p* = 0.29819	0.08, *p* = 0.4024	0.08, *p* = 0.42905
CENPA	0.03, *p* = 0.42363	−0.04, *p* = 0.69752	−0.03, *p* = 0.78042
ARC	−0.14, *p* = 0.08058	−0.02, *p* = 0.87338	−0.01, *p* = 0.9275
ERRFI1	−0.04, *p* = 0.08501	−0.03, *p* = 0.75554	−0.03, *p* = 0.70662
CSRNP1	−0.06, *p* = 0.56529	−0.03, *p* = 0.73185	−0.02, *p* = 0.77915
KDM6B	0.02, *p* = 0.43111	0, *p* = 0.98583	−0.01, *p* = 0.9362
EGR3	−0.08, *p* = 0.15211	0.1, *p* = 0.22062	0.1, *p* = 0.2386
TANC1	0.03, *p* = 0.58032	−0.02, *p* = 0.68021	−0.02, *p* = 0.71937
RASL10A	−0.07, *p* = 0.16861	0.01, *p* = 0.92154	0.01, *p* = 0.89461
SLC17A6	−0.03, *p* = 0.56269	−0.01, *p* = 0.86581	−0.02, *p* = 0.868
TAMALIN	−0.01, *p* = 0.7858	0.11, *p* = 0.23754	0.13, *p* = 0.18539
ZDHHC21	0.05, *p* = 0.11461	0.05, *p* = 0.56481	0.06, *p* = 0.54912
NR4A2	−0.08, *p* = 0.09242	0.01, *p* = 0.87624	0.04, *p* = 0.68243
OSBPL6	−0.12, *p* = 0.09218	−0.05, *p* = 0.52991	−0.06, *p* = 0.46194
DUSP4	−0.08, *p* = 0.08166	−0.07, *p* = 0.23798	−0.06, *p* = 0.2808
SSTR2	−0.04, *p* = 0.29886	0.04, *p* = 0.66881	0.04, *p* = 0.71109
NTN5	NA	0.01, *p* = 0.91139	0.02, *p* = 0.77849
CACNG5	0.03, *p* = 0.52488	−0.01, *p* = 0.91203	0, *p* = 0.9889
JUNB	0.01, *p* = 0.88626	−0.02, *p* = 0.77838	−0.02, *p* = 0.77732
FOS	−0.12, *p* = 0.08703	0.01, *p* = 0.93361	−0.01, *p* = 0.94578
PROX1	0.04, *p* = 0.08436	−0.03, *p* = 0.66585	−0.03, *p* = 0.71635
RASL11A	NA	−0.08, *p* = 0.24287	−0.09, *p* = 0.20206
PSMB5	−0.14, *p* = 0.09518	−0.05, *p* = 0.62883	−0.04, *p* = 0.67635
C1QL2	NA	−0.01, *p* = 0.90307	0, *p* = 0.99084
EGR4	−0.14, *p* = 0.02838	−0.05, *p* = 0.65832	−0.04, *p* = 0.758
C2CD4A	NA	−0.05, *p* = 0.62716	−0.07, *p* = 0.56111

Each of the 69 genes that are differentially expressed in the hippocampus of *Egr3−/−* vs WT mice at 1 h following ECS that were present in human schizophrenia study datasets are listed in column 1 (total = 58). Each column represents the fold change (FC) value of the human homolog in the listed schizophrenia study dataset. *p* values are adjusted for multiple comparisons.

To determine whether the 58 DEGs showed a statistically significant overlap with genes differentially expressed in the 13 schizophrenia studies, we conducted a gene set enrichment analysis using the hypeR package. These results revealed that the 58 DEGs we identified in the hippocampus of *Egr3−/−* compared with WT mice 1 h following ECS, of which homologs are present in the human datasets, are significantly enriched in the dataset from the study by Gandal and colleagues [40] (FDR = 0.021, Supplementary Fig. S5, Supplementary Table S10) This identified 18 overlapping genes between the two datasets (Supplementary Table S11).

Supplementary Fig. S6 shows the proportion of schizophrenia datasets in which each of the 58 DEGs identified in schizophrenia datasets was differentially expressed. The gene C1QL2 (complement C1q like 2) appeared in the greatest proportion of schizophrenia studies, followed by the IEG ARC. These findings support the hypothesis that EGR3 is required for expression of genes that are abnormally expressed in schizophrenia.

### EGR3-dependent DEGs are involved in DNA damage response

**Fig. 2:**
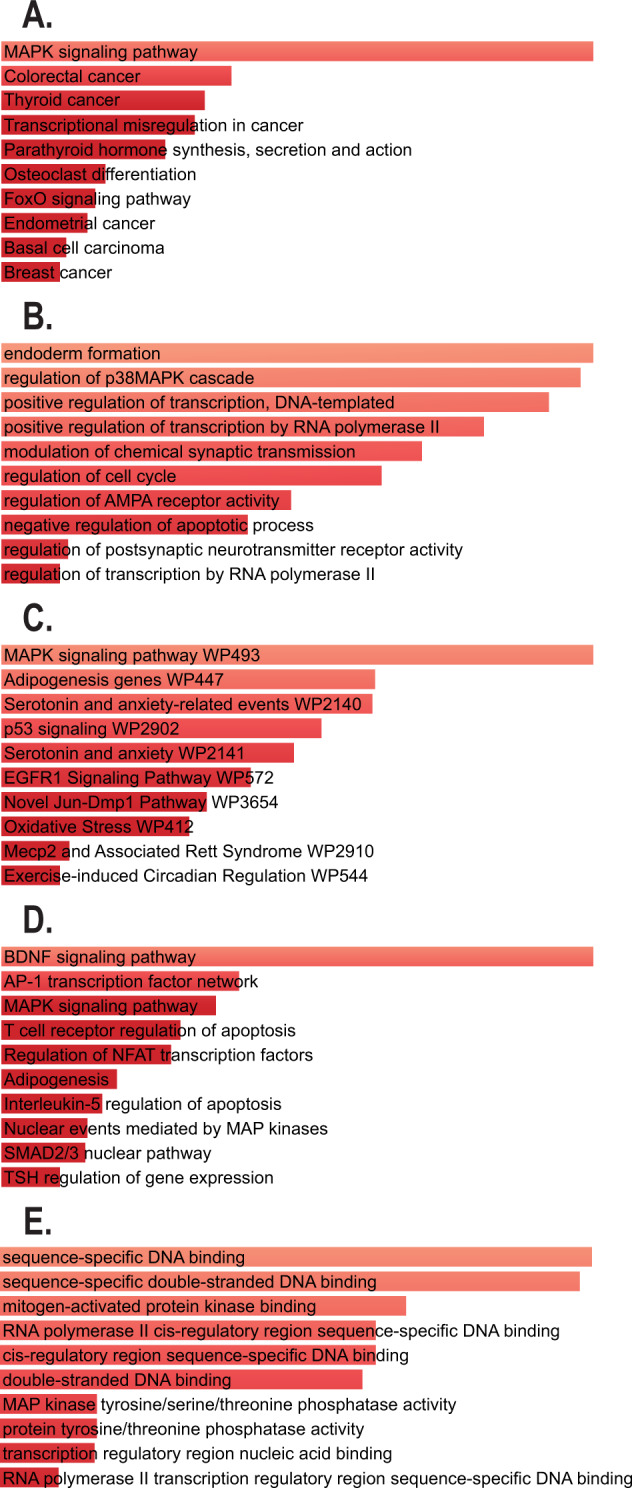
Top canonical pathways and biological function categories associated with DEGs generated by the Enrichr web tool. Enrichr pathway analyses performed on microarray results reveals biological processes associated with the genes that are differentially expressed in hippocampus of *Egr3−/−* mice compared to WT mice 1 h following ECS. The top 10 enriched pathways from each queried gene set library (**A** KEGG 2019 Mouse, **B** Gene Ontology (GO) Biological Process 2021, **C** WikiPathways 2019 Mouse, **D** BioPlanet 2019, and **E** GO Molecular Function 2021) are shown in order from most significant to least significant adjusted *p* value. The longer and lighter colored the bar, the more significant the pathway term. All pathways presented have significant adjusted *p* values < 0.05. One of the most significantly enriched pathways in the DEG list was MAPK signaling, specifically including GADD45B and GADD45G. Complete Enrichr results can be found in Supplementary Table S12.

To identify the major biological pathways regulated by EGR3 in the hippocampus, we conducted a canonical pathway analysis using Enrichr (Fig. 2). The results showed that one of the most significantly overrepresented pathways in the DEG list was MAPK signaling. One of the leading gene groups in this pathway and other top pathways was the GADD45 (Growth Arrest and DNA Damage) signaling genes (Fig. 2). A literature survey revealed that GADD45, as well as numerous of the top pathways we identified, including p53, ATM and Jak/STAT pathways, are involved in DNA damage response [44–46]. Based on these observations we chose to follow-up the microarray results with validation of genes that were relevant to DNA damage response.

### GADD45 signaling genes require *Egr3* for ECS-induced expression

The GADD45 family consists of proteins involved in regulation of DNA repair [47, 48], DNA demethylation [49], neurogenesis [50] and response to stress [51]. Since GADD45 signaling was significantly overrepresented in the DEG list, we conducted follow up studies on the members of the GADD45 signaling pathway that were in our DEG list: *Gadd45b* and *Gadd45g*.

**Fig. 3:**
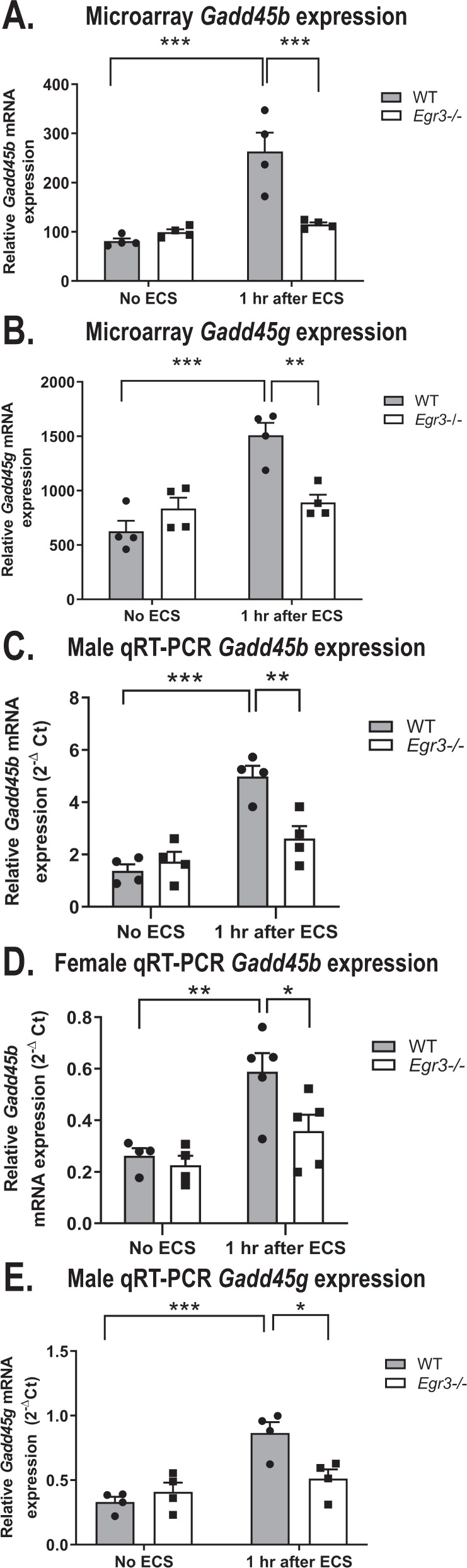
GADD45 family genes are differentially expressed in *Egr3*−/− mice. **A**, **B** Expression microarray results of GADD45B pathway gene expression in hippocampus from WT and *Egr3−/−* mice at baseline (No ECS) and 1 h after ECS. **A** *Gadd45b*, **B** *Gadd45g*. Quantitative RT-PCR validation of *Gadd45b* in (**C**) the original male cohort and (**D**) the replication female cohort. **E** qRT-PCR validation of *Gadd45g* results in original male cohort. (For experiments in **A**–**C** and **E** *n* = 4 animals/group, experiments in **D** WT: No ECS, *n* = 4; 1 h after ECS, *n* = 5; *Egr3−/−*: No ECS, *n* = 4; 1 h after ECS, *n* = 5; *p < 0.05, **p < 0.01, ***p < 0.001, controlled for multiple comparisons, values represent means ± SEM). Statistical analyses for these, and all subsequent graphs, are shown in Table 3.

Expression microarray results showed that ECS causes a ≥ 2-fold increase in mRNA levels of both of these genes in WT mice that was not present in *Egr3−/−* mice (Fig. 3A, B). For both of these genes this resulted in a significantly lower level of expression following ECS in *Egr3−/−* mice than in WT controls (Table 3 lists results of ANOVAs and post-hoc comparisons for all follow-up studies.)

**Table 3:** Statistics for microarray and qRT-PCR analyses.

Gene	Two-way ANOVA results	Post hoc comparison (Tukey’s test) results (*p* < 0.05, **p < 0.01, ***p < 0.001, ****p < 0.0001)
Gadd45b (M, microarray)	Sig interaction of genotype & treatment F (1, 12) = 18.39, *p* = 0.0011	WT vs WT ECS ***, WT ECS vs KO ECS ***
Gadd45b (M, qRT PCR)	Sig interaction of genotype & treatment F (1, 12) = 12.55, *p* = 0.0041	WT vs WT ECS ***, WT ECS vs KO ECS **
Gadd45b (F, qRT PCR)	Sig effect of genotype F (1, 14) = 5.302, *p* = 0.0372; Sig effect of treatment F (1, 14) = 15.80, *p* = 0.0014	WT vs WT ECS **, WT ECS vs KO ECS *
Gadd45g (M, microarray)	Sig interaction of genotype & treatment F (1, 12) = 18.39, *p* = 0.0011	WT vs WT ECS ***, WT ECS vs KO ECS **
Gadd45g (M, qRT PCR)	Sig interaction of genotype & treatment F (1, 12) = 9.875, *p* = 0.0085	WT vs WT ECS ***, WT ECS vs KO ECS *
Gadd45g (F, qRT PCR)	Not sig	N/A
Cdnk1a (M, microarray)	Sig interaction of genotype & treatment F (1, 12) = 7.316, *p* = 0.0191	WT vs WT ECS ****, KO vs KO ECS *, WT ECS vs KO ECS *
Cenpa (M, microarray)	Sig interaction of genotype & treatment F (1, 12) = 263.1, *p* < 0.0001	WT vs WT ECS ****, WT ECS vs KO ECS ****
Cenpa (M, qRT PCR)	Sig interaction of genotype & treatment F (1, 12) = 103.9, *p* < 0.0001	WT vs WT ECS ****, WT ECS vs KO ECS ****
Cenpa (F, qRT PCR)	Sig interaction of genotype & treatment F (1, 14) = 68.28, *p* < 0.0001	WT vs WT ECS ****, WT ECS vs KO ECS ****
Fos (M, microarray)	Sig interaction of genotype & treatment F (1, 12) = 10.92, *p* = 0.0063	WT vs WT ECS ****, KO vs KO ECS *, WT ECS vs KO ECS**
Fos (M, qRT PCR)	Sig interaction of genotype & treatment F (1, 12) = 5.808, *p* = 0.0329	WT vs WT ECS **, WT ECS vs KO ECS *
Fos (F, qRT PCR)	Sig effect of treatment F (1, 14) = 8.392, *p* = 0.0117	WT vs WT ECS *
Fosb (M, microarray)	Sig interaction of genotype & treatment F (1, 12) = 63.79, *p* < 0.0001	WT vs WT ECS ****, WT ECS vs KO ECS ****
Fosb (M, qRT PCR)	Sig interaction of genotype & treatment F (1, 12) = 14.01, *p* = 0.0028	WT vs WT ECS ***, WT ECS vs KO ECS ***
Fosb (F, qRT PCR)	Sig interaction of genotype & treatment F (1, 14) = 7.094, *p* = 0.0185	WT vs WT ECS ***, WT ECS vs KO ECS **
Junb (M, microarray)	Sig interaction of genotype & treatment F (1, 12) = 18.22, *p* = 0.0011	WT vs WT ECS ****, KO vs KO ECS *, WT ECS vs KO ECS***
Junb (M, qRT PCR)	Sig effect of treatment F (1, 12) = 6.247, *p* = 0.0280	Posthoc comparisons NS
Junb (F, qRT PCR)	Sig effect of treatment F (1, 14) = 6.569, *p* = 0.0225; sig effect of genotype F (1, 14) = 4.608, *p* = 0.0498	Posthoc comparisons NS
Nr4a2 (M, microarray)	Sig interaction of genotype & treatment F (1, 12) = 15.90, *p* = 0.0018	WT vs WT ECS ***, WT ECS vs KO ECS ***
Nr4a2 (M, qRT PCR)	Sig interaction of genotype & treatment F (1, 12) = 7.050, *p* = 0.0210	WT vs WT ECS ***, WT ECS vs KO ECS **
Nr4a2 (F, qRT PCR)	Sig interaction of genotype & treatment F (1, 14) = 5.230, *p* = 0.0383	WT vs WT ECS *
Mef2c (M, microarray)	Sig interaction of genotype & treatment F (1, 12) = 21.07, *p* = 0.0006	WT vs WT ECS**, KO vs. WT ECS**
Mef2c (M, qRT PCR)	Sig interaction of genotype & treatment F (1, 12) = 9.484, *p* = 0.0095	Posthoc comparisons NS
Mef2c (F, qRT PCR)	Not sig	N/A
Calb2 (M, microarray)	Sig effect of genotype F (1, 12) = 48.99, *p* < 0.0001	WT vs KO***, WT ECS vs. KO ECS**
Calb2 (M, qRT PCR)	Sig effect of genotype F (1, 12) = 11, *p* = 0.0061	Posthoc comparisons NS
Calb2 (F, qRT PCR)	Not sig	N/A

To validate these findings, we conducted qRT-PCR on the original mRNA samples that were used to perform the microarray analysis. Since the animals used in the microarray were all male, we replicated the study in female animals to determine if the gene expression changes were sex specific.

Expression of *Gadd45b* validated in the mRNA samples used for the microarray (Fig. 3C). In addition, in the female validation group *Gadd45b* showed the same pattern of a ≥2-fold increase in expression following ECS in WT mice that was absent in *Egr3−/−* mice (Fig. 3D). For *Gadd45g*, the microarray findings were validated in the male mRNA samples (Fig. 3E) but did not show a significant difference between genotypes in the female replication cohort (data not shown, two-way ANOVA not significant, see Table 3). These results indicate that *Egr3* is required for activity-dependent upregulation of GADD45 family gene expression in the hippocampus of male mice and, in the case of *Gadd45b*, also in female animals.

### DNA damage response gene *Cenpa* upregulated 12-fold by ECS, which requires EGR3

**Fig. 4:**
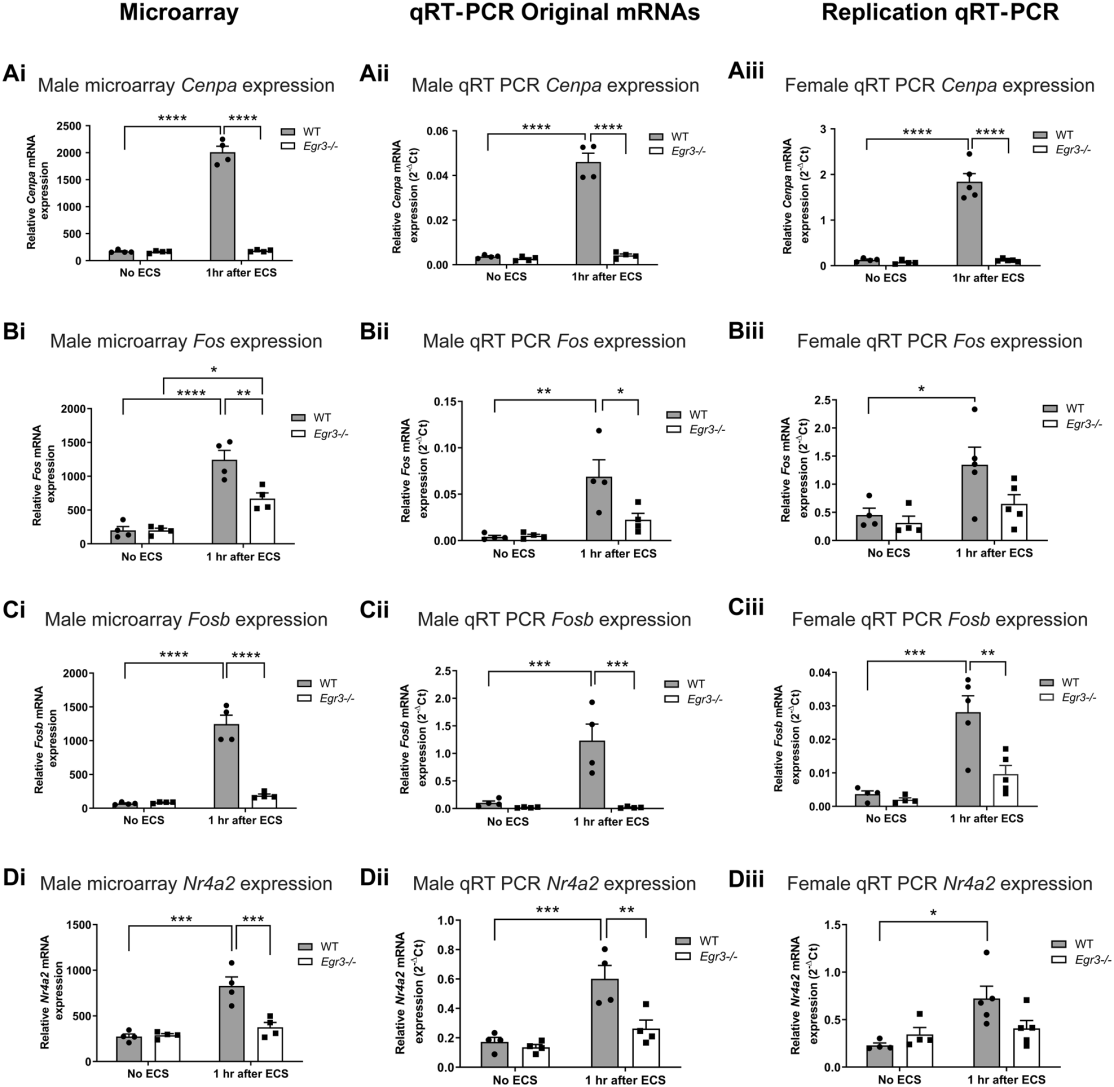
Numerous genes are differentially expressed in *Egr3*−/− mice compared with WT mice following ECS. Microarray analysis results (i, *n* = 4 animals/group) and follow-up qRT-PCR results performed on the original RNA samples used in the microarray (ii, *n* = 4 animals/group) and in a replication cohort of female mice (iii, WT: No ECS, *n* = 4; 1 h after ECS, *n* = 5; *Egr3−/−*: No ECS, *n* = 4; 1 h after ECS, *n* = 5). **A** *Cenpa*, the most highly DEG in the dataset, AP-1 family genes **B** *Fos* and **C** *Fosb*, and additional memory-related gene **D** *Nr4a2*, which is also involved in DNA repair. (*p < 0.05, **p < 0.01, ***p < 0.001, ****p < 0.0001, controlled for multiple comparisons, values represent means ± SEM).

Histone H3-like centromere protein A (CENPA), a protein essential for the initial stages of centromere assembly [52], is critical for efficient DNA repair in vivo [53]*. Cenpa* showed the greatest degree of differential expression between *Egr3−/−* and WT mice of all the genes in the microarray dataset. This was due to a 12-fold upregulation of *Cenpa* in WT mice in response to ECS that was entirely absent in *Egr3−/−* mice (Fig. 4Ai, Table 3). This finding was validated by qRT-PCR in male mRNAs as well in the female replication cohort, in which ECS induced a 13-fold and 15-fold increase in *Cenpa* expression (respectively) in WT mice with no change in expression in *Egr3−/−* mice (Fig. 4Aii, Aiii, Table 3).

### ECS-induced gene expression of AP1 transcription factor components *Fos* and *Fosb* is *Egr3* dependent

The Activator protein 1 (AP1) transcription factor is a dimeric transcription factor whose subunits belong to four different families of DNA-binding proteins including the *Jun* family, *Fos* family, ATF/cyclic AMP-responsive element-binding (CREB), and the musculoaponeurotic fibrosarcoma (*Maf*) family [54]. The AP-1 transcription factor components play critical roles in cancer [55], immune system function [56], neurite growth [57], and DNA repair [58]. Results from the microarray showed that three AP-1 components *Fos*, *Fosb*, and *Jun* showed ECS-induced expression in WT mice that was either significantly lower, or absent, in *Egr3−/−* mice after ECS (Fig. 4Bi, Ci, Table 3). Both *Fos* and *Fosb* findings were validated in male RNA samples (Fig. 4Bii, Cii, Table 3) and replicated in the female cohort by qRT-PCR (Fig. 4Biii, Ciii, Table 3).

The initial microarray showed differential expression of *Jun* between *Egr3−/−* and WT mice. The follow-up qRT-PCR studies revealed a significant effect of genotype in the female replication cohort, but not in the male validation study (Table 3).

### ECS-mediated induction of memory regulation gene *Nr4a2* is *Egr3-*dependent

In addition to genes involved in DNA damage response, based on our Enrichr analysis results, the next category of genes that we decided to investigate further were those involved in nervous system function and regulation of behavior. The gene nuclear receptor subfamily 4 member 2 (*Nr4a2*) particularly stood out due to its roles in memory and neurophysiologic processes. *Nr4a2* is required for both long-term memory and object recognition [59], hippocampal neurogenesis [60], and was recently shown to be critical for DNA repair in vitro [61].

In WT mice, ECS induced a 2.8–3-fold upregulation of *Nr4a2* expression that was absent in *Egr3−/−* mice (Fig. 4Di, Table 3). The results of the microarray were validated in the male RNAs and replicated in the female cohort by qRT-PCR (Fig. 4Dii, Diii, Table 3).

### Genes upregulated in *Egr3−/−* mice including *Mef2c* and *Calb2* are linked to schizophrenia

The majority of genes we chose for validation studies were upregulated in WT mice after ECS compared to *Egr3−/−* mice following ECS. However, a small number of genes showed increased expression in *Egr3−/−* mice compared with WT mice after ECS. We chose two genes from this group for validation studies based on their degree of fold-change induction, involvement in behavior and/or DNA damage response, and association with schizophrenia. The first, transcription factor myocyte enhancer factor 2c (*Mef2c*), is a key regulator of learning and memory in vivo [62]. The second, calbindin 2 (*Calb2*), encodes the protein calretinin, a critical regulator of long-term potentiation in the dentate gyrus [63]. Previous studies showed that enrichment of MEF2C motifs were found in sequences surrounding the top single nucleotide polymorphisms within schizophrenia risk loci [64], and increased levels of calretinin were reported in the dentate gyrus of schizophrenia and bipolar patients compared to controls [65].

**Fig. 5:**
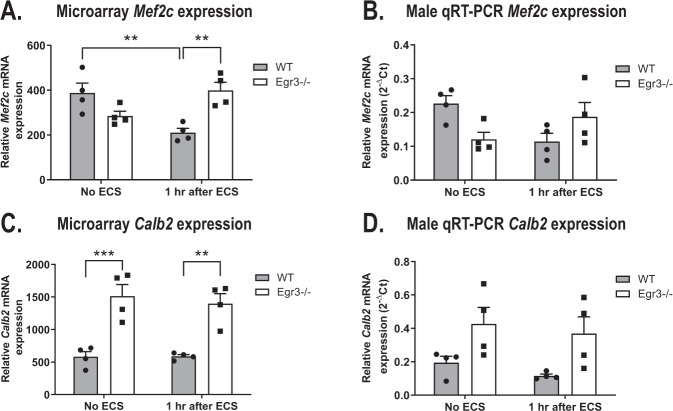
*Mef2c* and *Calb2* display unique patterns of regulation. Microarray analysis results (**A**, **C**) and follow-up qRT-PCR results in males (**B**, **D**) for schizophrenia associated gene *Mef2c* (**A**, **B**) and *Calb2* (**C**, **D**), genes overexpressed in schizophrenia. (*n* = 4 animals/group; ANOVAs were significant for each (see Table 3), *p*-values represent post-hoc analyses, **p < 0.01, ***p < 0.001, controlled for multiple comparisons, values represent means ± SEM).

The *Mef2c* microarray results showed that ECS caused a decrease in *Mef2c* expression in WT mice which did not occur in *Egr3−/−* mice (Fig. 5A, Table 3). The opposing effect of ECS on *Mef2c* expression resulted in a significant interaction between ECS treatment and genotype in the two-way ANOVA in the microarray, a result that was validated by qRT-PCR in the male mRNAs (Fig. 5A, B, Table 3). In contrast, in females no significant changes in *Mef2c* expression were seen (Table 3).

The *Calb2* microarray results showed a pattern that was uncommon in the dataset. Expression of *Calb2* is significantly reduced in WT mice compared to *Egr3−/−* mice at baseline and does not change in response to ECS in either genotype. This pattern, which was validated by qRT-PCR in males (Fig. 5C, D, Table 3), was not significant in the female qRT-PCR results (Table 3).

## Discussion

Major advances in genetics and genomics over the last decade and a half have led to identification of hundreds of genes associated with risk for neuropsychiatric and neurodegenerative disorders. One method to identify mechanisms that unite these findings, and thus underlie illness etiology, is to identify the “master regulatory genes” that orchestrate expression of large numbers of these disease-associated genes. *EGR3* has been identified as such a master regulator in neuropsychiatric illnesses of schizophrenia, bipolar disorder and, most recently, Alzheimer’s dementia [2–4]. These studies have relied on bioinformatics resources to identify the gene interaction relationships that led to these discoveries. However, few studies have validated the genes that require *Egr3* for their expression in the brain in vivo. Our findings reveal numerous genes that are dependent upon *Egr3* for their normal expression in response to neuronal activity in the mouse hippocampus. Many of these genes either map to schizophrenia GWAS risk loci or have been identified as differentially regulated in schizophrenia studies.

As an immediate early gene transcription factor, *Egr3* is rapidly expressed in the brain in response to neuronal activity and, in turn, regulates the subsequent set of genes expressed in response to that activity. *Egr3* is thus poised to translate environmental stimuli into changes in gene expression that dictate the brain’s response to the outside world. Our studies in mice identified the critical roles of *Egr3* in stress-responsive behavior, memory, and synaptic plasticity [13, 15]. Based on these findings, and the upstream signaling events that trigger *EGR3* expression, we hypothesized that dysfunction of *EGR3* would disrupt the brain’s resilient response to stress, resulting in neuropathology which, over time, may give rise to symptoms of neuropsychiatric illness [13, 15, 16]. Although *EGR3* itself is not located at one of the 145 loci associated with risk for schizophrenia, each of the other members of the *EGR* family, *EGR1*, *EGR2*, *EGR4*, as well as the *EGR* co-regulatory binding factor *NAB2* each, map to schizophrenia GWAS loci [19–21], and *EGR3* interacts in a co-regulatory feedback loop with *EGR1*, *EGR2*, and *NAB2* [18].

Our hypothesis has subsequently been supported by studies showing both genetic association of *EGR3* with schizophrenia [9, 66–68], as well as decreased *EGR3* gene expression in brains of schizophrenia patients [66, 69] and fibroblasts isolated from schizophrenia patients [70]. Recent in silico studies identified *EGR3* as a central gene in a network of transcription factors and microRNAs associated with schizophrenia risk [2], a master regulator of genes that are differentially regulated in bipolar disorder patients [3], and a critical regulator of DEGs involved in synaptic function in Alzheimer’s disease [4]. In total, these findings suggest that altered *EGR3* activity, or disruption of proteins that function upstream or downstream of *EGR3*, may increase risk for neuropsychiatric disorders and play a role of development of neurodegenerative disease.

To identify *Egr3*-dependent genes in the brain, we used ECS to maximally induce immediate early gene expression in the hippocampi of WT and *Egr3−/−* mice and identified DEGs using a microarray-based approach. We found genes involved in regulation of behavior and DNA damage response pathways to be significantly overrepresented in our DEG list. Our results suggest that *Egr3* is necessary for ECS-dependent stimulation of a subset of genes involved in regulation of nervous system function including regulation of memory (*Nr4a2* [59]), neurogenesis and synaptic plasticity (*Gadd45b* [50]*, Bdnf* [71]), behavior (*Fos* [72], *Fosb* [73]) and DNA damage response, particularly, DNA repair (*Cenpa* [53], GADD45 family proteins Gadd45b and *Gadd45g* [47, 48], *Fos* and *Fosb* that are part of the AP-1 transcription factor [58], and *Nr4a2* [61]). We also report that two genes that show elevated expression in schizophrenia (*Calb2* [65]*, Mef2c* [64]) are upregulated in mice lacking *Egr3*. In total, our findings suggest that *Egr3* is critical for the normal activity-responsive expression of genes involved in brain function and the DNA damage response.

### The importance of DNA repair in neurons, findings of DNA damage regulating genes involved in behavior

In neurons, DNA damage can occur during normal cellular activity and in processes involving DNA replication, such as neurogenesis [74]. Neurons are postmitotic, and typically cannot be replaced by new cells if DNA damage reaches critical levels. Therefore, neurons rely heavily on effective DNA repair mechanisms to maintain homeostasis [75]. DNA damage is often associated with aging and disease pathology; however, two recent paradigm-shifting studies highlight the role of DNA damage in regulation of normal physiological function in neurons. The first study, by Suberbeille and colleagues [76], showed that neuronal activity triggered by exploration of a novel environment can cause DNA damage in the form of DNA double-stranded breaks in the cortex and hippocampus of young adult WT mice. The second study, by Madabhushi and colleagues, demonstrated that in vitro stimulation of primary neurons induced DNA double-stranded breaks in the promoters of immediate early genes that was essential for their activity-dependent induction [77]. They also showed that inhibiting non-homologous end joining, a DNA repair pathway, caused a sustained “switched on” state of gene expression perturbing the normal temporal dynamics of immediate early gene expression.

We found several genes from the Madabhushi study to be differentially expressed in our results. These included *Fos*, *Fosb*, and *Nr4a2*, which failed to be induced in *Egr3−/−* mice after ECS, and represented 3 of the 12 genes that showed upregulation in the Madabhushi et al. study following etoposide treatment of neurons [77]. Madabhushi and colleagues also reported that of these genes, *Fos* and *Fosb* showed enrichment of DNA damage double strand breaks (γ-H2AX) in their promoters and were induced following neuronal activity in vitro [77].

We show that *Egr3* is necessary for the neuronal activity-induced expression of these genes. Given the roles of these genes in regulation of neuronal function, impaired expression of these genes may contribute to the behavioral and cognitive deficits seen in *Egr3−/−* mice [13, 78]. In addition to playing a role in behavior regulation, *Fos* and *Fosb* are members of the AP-1 transcription factor complex, a critical regulator of DNA repair genes [58]. In line with these data, we also found that genes belonging to the GADD45 signaling pathway, a major DNA damage response pathway [47, 48], showed impaired induction in *Egr3−/−* mice following ECS. GADD45b was recently identified as an EGR3-dependent gene in prostate cancer, and EGR3 was shown to bind to the GADD45B promoter in vivo and upregulate expression of GADD45B in vitro [79].

In addition, other DNA damage response genes including *Cenpa* and *Nr4a2*, recently found to play a role in DNA repair [53, 61] showed a similar lack of induction in the *Egr3−/−* mice after undergoing ECS. For several of these genes we saw particularly robust induction following ECS in wildtype mice including a 12-fold induction for *Cenpa* and a 15-fold induction for *Fos* that the *Egr3−/−* mice lacked. Given that *EGR3* is induced in response to DNA damaging stimuli in vitro [80], our findings suggest that lack of functional *Egr3* results in diminished activation of genes involved in DNA damage response. This dysfunction of *Egr3* may increase susceptibility to DNA damage, impacting normal physiological activation of genes and contributing to increased DNA damage observed in neuropsychiatric illness [81–85].

### Genes upregulated in *Egr3−/−* mice

While the majority of DEGs in our data failed to be induced by ECS in *Egr3−/−* mice, a small subset showed the opposite trend. Key among these genes were *Mef2c* and *Calb2*, which showed the most profound increase in *Egr3−/−* mice following ECS compared to WT mice. Prior studies show that both of these genes show increased expression in schizophrenia patients’ brains compared to controls; *Mef2c* in the prefrontal cortex [64] and *Calb2* in the dentate gyrus [65]. A previous study showed that deletion of *Mef2c* impairs hippocampal-dependent learning and memory in vivo [62]. Also, *Mef2c* limits excessive synapse formation during activity-dependent synaptogenesis in the dentate gyrus [62]. Mice lacking the *Calb2* encoded protein calretinin, show impaired hippocampal long-term potentiation (LTP) [86]. Studies suggest that temporal and spatial regulation of MEF2 family members [87] and *Calb2* [88] is essential for normal brain development.

Both timing and level of immediate early gene expression are critical features of their function. Either insufficient expression, or persistent overexpression, of immediate early genes, or the factors that regulate them, can negatively affect learning [89], or cause anxiety-like behavior, respectively [90]. Our findings indicate that *Egr3* may influence the temporal regulation of these genes, where a lack of *Egr3* may lead to a perpetual “switched on” state of gene expression for genes such as *Mef2c* and *Calb2* that is not observed in WT mice, and may negatively impact normal brain function.

The current study has several limitations. First, seizure induces maximal expression of activity-dependent genes. Further studies will be important to determine whether the DEGs identified in our study following ECS are also regulated by physiologic stimuli such as exposure to a novel environment. A second limitation is that the *Egr3−/−* mice used in this study are “conventional knockout mice” and lack normal expression of *Egr3* from embryogenesis throughout life [30]. Therefore, the gene expression differences identified in our study may result from abnormal hippocampal development. In addition, the timing of the changes in gene expression in the current study are seen before maximal EGR3 protein expression, which continues to increase until at least 4 h after ECS [91]. Therefore, the DEGs identified may not be directly regulated by EGR3. Finally, the *Egr3* gene is disrupted in *Egr3−/−* mice by removal of the DNA binding domain. Although *Egr3−/−* mice lack WT protein expression, the possibility that a truncated form of EGR3 protein is expressed, which may have biological effects, cannot be ruled out [30].

In summary, we report activity-dependent gene expression changes in the hippocampus of *Egr3−/−* mice, previously shown to exhibit schizophrenia-like behavioral abnormalities and memory deficits [13, 15]. We have validated numerous genes that were differentially expressed in the microarray data using qRT-PCR in the original male RNAs and a replication cohort of female mice, demonstrating that many of these effects are independent of sex while others appear sex-dependent. These genes are involved in behavior and DNA damage response, with a subset playing dual roles in neuronal function and DNA repair including *Gadd45b*, *Nr4a2* and *Bdnf*. Further studies are needed to define the role of EGR3 in regulating DNA response genes necessary to repair DNA double strand breaks induced by neuronal activity. In conclusion, our studies demonstrate that EGR3 is a critical dual regulator of behavior and DNA damage response genes, and further define its role in brain function and neuropsychiatric and neurodegenerative illnesses characterized by cognitive dysfunction. The identification of EGR3-dependent genes in the mouse hippocampus may help to explain findings indicating that EGR3 may be a master regulator of genes differentially expressed in neuropsychiatric illnesses ranging from schizophrenia and bipolar disorder to Alzheimer’s dementia [2–4].

## Supplementary information

Supplemental Figure 1

Supplemental Figure 2

Supplemental Figure 3

Supplemental Figure 4

Supplemental Figure 5

Supplemental Figure 6

Supplemental Tables 1–3

Supplemental Table 4

Supplemental Table 5

Supplemental Table 6

Supplemental Table 7

Supplemental Table 8

Supplemental Table 9

Supplemental Table 10

Supplemental Table 11

Supplemental Table 12

## References

[1] Brainstorm C, Anttila V, Bulik-Sullivan B, Finucane HK, Walters RK, Bras J (2018). Analysis of shared heritability in common disorders of the brain. Science.

[2] Guo AY, Sun J, Jia P, Zhao Z (2010). A novel microRNA and transcription factor mediated regulatory network in schizophrenia. BMC Syst Biol.

[3] Pfaffenseller B, da Silva Magalhaes PV, De Bastiani MA, Castro MA, Gallitano AL, Kapczinski F (2016). Differential expression of transcriptional regulatory units in the prefrontal cortex of patients with bipolar disorder: potential role of early growth response gene 3. Transl Psychiatry.

[4] Canchi S, Raao B, Masliah D, Rosenthal SB, Sasik R, Fisch KM (2019). Integrating gene and protein expression reveals perturbed functional networks in Alzheimer’s disease. Cell Rep.

[5] Li L, Carter J, Gao X, Whitehead J, Tourtellotte WG (2005). The neuroplasticity-associated arc gene is a direct transcriptional target of early growth response (Egr) transcription factors. Mol Cell Biol.

[6] Kirov G, Pocklington AJ, Holmans P, Ivanov D, Ikeda M, Ruderfer D (2012). De novo CNV analysis implicates specific abnormalities of postsynaptic signalling complexes in the pathogenesis of schizophrenia. Mol Psychiatry.

[7] Fromer M, Pocklington AJ, Kavanagh DH, Williams HJ, Dwyer S, Gormley P (2014). De novo mutations in schizophrenia implicate synaptic networks. Nature.

[8] Purcell SM, Moran JL, Fromer M, Ruderfer D, Solovieff N, Roussos P (2014). A polygenic burden of rare disruptive mutations in schizophrenia. Nature.

[9] Huentelman MJ, Muppana L, Corneveaux JJ, Dinu V, Pruzin JJ, Reiman R (2015). Association of SNPs in EGR3 and ARC with schizophrenia supports a biological pathway for schizophrenia risk. PloS One.

[10] Roberts DS, Hu Y, Lund IV, Brooks-Kayal AR, Russek SJ (2006). Brain-derived neurotrophic factor (BDNF)-induced synthesis of early growth response factor 3 (Egr3) controls the levels of type A GABA receptor alpha 4 subunits in hippocampal neurons. J Biol Chem.

[11] Roberts DS, Raol YH, Bandyopadhyay S, Lund IV, Budreck EC, Passini MA (2005). Egr3 stimulation of GABRA4 promoter activity as a mechanism for seizure-induced up-regulation of GABA(A) receptor alpha4 subunit expression. Proc Natl Acad Sci USA.

[12] Riley JD, Delahunty C, Alsadah A, Mazzola S, Astbury C (2020). Further evidence of GABRA4 and TOP3B as autism susceptibility genes. Eur J Med Genet.

[13] Gallitano-Mendel A, Izumi Y, Tokuda K, Zorumski CF, Howell MP, Muglia LJ (2007). The immediate early gene early growth response gene 3 mediates adaptation to stress and novelty. Neuroscience.

[14] Olney JW, Newcomer JW, Farber NB (1999). NMDA receptor hypofunction model of schizophrenia. J Psychiatr Res.

[15] Gallitano-Mendel A, Wozniak DF, Pehek EA, Milbrandt J (2008). Mice lacking the immediate early gene Egr3 respond to the anti-aggressive effects of clozapine yet are relatively resistant to its sedating effects. Neuropsychopharmacology.

[16] Marballi KK, Gallitano AL (2018). Immediate early genes anchor a biological pathway of proteins required for memory formation, long-term depression and risk for schizophrenia. Front Behav Neurosci.

[17] Yamagata K, Kaufmann WE, Lanahan A, Papapavlou M, Barnes CA, Andreasson KI (1994). Egr3/Pilot, a zinc finger transcription factor, is rapidly regulated by activity in brain neurons and colocalizes with Egr1/zif268. Learn Mem.

[18] Kumbrink J, Kirsch KH, Johnson JP (2010). EGR1, EGR2, and EGR3 activate the expression of their coregulator NAB2 establishing a negative feedback loop in cells of neuroectodermal and epithelial origin. J Cell Biochem.

[19] Pardinas AF, Holmans P, Pocklington AJ, Escott-Price V, Ripke S, Carrera N (2018). Common schizophrenia alleles are enriched in mutation-intolerant genes and in regions under strong background selection. Nat Genet.

[20] Schizophrenia Working Group of the Psychiatric Genomics C. (2014). Biological insights from 108 schizophrenia-associated genetic loci. Nature.

[21] Trubetskoy V, Pardinas AF, Qi T, Panagiotaropoulou G, Awasthi S, Bigdeli TB (2022). Mapping genomic loci implicates genes and synaptic biology in schizophrenia. Nature.

[22] Liu D, Evans I, Britton G, Zachary I (2008). The zinc-finger transcription factor, early growth response 3, mediates VEGF-induced angiogenesis. Oncogene.

[23] Jones MW, Errington ML, French PJ, Fine A, Bliss TV, Garel S (2001). A requirement for the immediate early gene Zif268 in the expression of late LTP and long-term memories. Nat Neurosci.

[24] Nagarajan R, Svaren J, Le N, Araki T, Watson M, Milbrandt J (2001). EGR2 mutations in inherited neuropathies dominant-negatively inhibit myelin gene expression. Neuron.

[25] Meyers KT, Marballi KK, Brunwasser SJ, Renda B, Charbel M, Marrone DF (2018). The immediate early gene Egr3 is required for hippocampal induction of bdnf by electroconvulsive stimulation. Front Behav Neurosci.

[26] Hastings KT, Elizalde D, Muppana L, Levine S, Kamel CM, Ingram WM (2017). Nab2 maintains thymus cellularity with aging and stress. Mol Immunol.

[27] Suehiro J, Hamakubo T, Kodama T, Aird WC, Minami T (2010). Vascular endothelial growth factor activation of endothelial cells is mediated by early growth response-3. Blood.

[28] Li S, Miao T, Sebastian M, Bhullar P, Ghaffari E, Liu M (2012). The transcription factors Egr2 and Egr3 are essential for the control of inflammation and antigen-induced proliferation of B and T cells. Immunity.

[29] Weigelt K, Carvalho LA, Drexhage RC, Wijkhuijs A, de Wit H, van Beveren NJ (2011). TREM-1 and DAP12 expression in monocytes of patients with severe psychiatric disorders. EGR3, ATF3 and PU.1 as important transcription factors. Brain Behav Immun.

[30] Tourtellotte WG, Milbrandt J (1998). Sensory ataxia and muscle spindle agenesis in mice lacking the transcription factor Egr3. Nat Genet.

[31] Reich M, Liefeld T, Gould J, Lerner J, Tamayo P, Mesirov JP (2006). GenePattern 2.0. Nat Genet.

[32] Wescombe L, Lahooti H, Gopinath B, Wall JR (2010). The cardiac calsequestrin gene (CASQ2) is up-regulated in the thyroid in patients with Graves’ ophthalmopathy-support for a role of autoimmunity against calsequestrin as the triggering event. Clin Endocrinol.

[33] Xie Z, Bailey A, Kuleshov MV, Clarke DJB, Evangelista JE, Jenkins SL (2021). Gene set knowledge discovery with enrichr. Curr Protoc.

[34] Federico A, Monti S (2020). hypeR: an R package for geneset enrichment workflows. Bioinformatics.

[35] Maple AM, Zhao X, Elizalde DI, McBride AK, Gallitano AL (2015). Htr2a Expression Responds Rapidly to Environmental Stimuli in an Egr3-Dependent Manner. ACS Chem Neurosci.

[36] Schmittgen TD, Livak KJ (2008). Analyzing real-time PCR data by the comparative C(T) method. Nat Protoc.

[37] Xu Y, Yao Shugart Y, Wang G, Cheng Z, Jin C, Zhang K (2016). Altered expression of mRNA profiles in blood of early-onset schizophrenia. Sci Rep.

[38] Wu X, Shukla R, Alganem K, Zhang X, Eby HM, Devine EA (2021). Transcriptional profile of pyramidal neurons in chronic schizophrenia reveals lamina-specific dysfunction of neuronal immunity. Mol Psychiatry.

[39] Wen Z, Nguyen HN, Guo Z, Lalli MA, Wang X, Su Y (2014). Synaptic dysregulation in a human iPS cell model of mental disorders. Nature.

[40] Gandal MJ, Haney JR, Parikshak NN, Leppa V, Ramaswami G, Hartl C (2018). Shared molecular neuropathology across major psychiatric disorders parallels polygenic overlap. Science.

[41] Hoffman GE, Hartley BJ, Flaherty E, Ladran I, Gochman P, Ruderfer DM (2017). Transcriptional signatures of schizophrenia in hiPSC-derived NPCs and neurons are concordant with post-mortem adult brains. Nat Commun.

[42] Roussos P, Katsel P, Davis KL, Siever LJ, Haroutunian V (2012). A system-level transcriptomic analysis of schizophrenia using postmortem brain tissue samples. Arch Gen Psychiatry.

[43] Torrey EF, Webster M, Knable M, Johnston N, Yolken RH (2000). The stanley foundation brain collection and neuropathology consortium. Schizophrenia Res.

[44] Jin S, Mazzacurati L, Zhu X, Tong T, Song Y, Shujuan S (2003). Gadd45a contributes to p53 stabilization in response to DNA damage. Oncogene.

[45] Blackford AN, Jackson SP (2017). ATM, ATR, and DNA-PK: the trinity at the heart of the DNA damage response. Mol Cell.

[46] Barry SP, Townsend PA, Knight RA, Scarabelli TM, Latchman DS, Stephanou A (2010). STAT3 modulates the DNA damage response pathway. Int J Exp Pathol.

[47] Smith ML, Ford JM, Hollander MC, Bortnick RA, Amundson SA, Seo YR (2000). p53-mediated DNA repair responses to UV radiation: studies of mouse cells lacking p53, p21, and/or gadd45 genes. Mol Cell Biol.

[48] Smith ML, Chen IT, Zhan Q, Bae I, Chen CY, Gilmer TM (1994). Interaction of the p53-regulated protein Gadd45 with proliferating cell nuclear antigen. Science.

[49] Barreto G, Schafer A, Marhold J, Stach D, Swaminathan SK, Handa V (2007). Gadd45a promotes epigenetic gene activation by repair-mediated DNA demethylation. Nature.

[50] Ma DK, Jang MH, Guo JU, Kitabatake Y, Chang ML, Pow-Anpongkul N (2009). Neuronal activity-induced Gadd45b promotes epigenetic DNA demethylation and adult neurogenesis. Science.

[51] Takekawa M, Saito H (1998). A family of stress-inducible GADD45-like proteins mediate activation of the stress-responsive MTK1/MEKK4 MAPKKK. Cell.

[52] Hoffmann S, Dumont M, Barra V, Ly P, Nechemia-Arbely Y, McMahon MA (2016). CENP-A is dispensable for mitotic centromere function after initial centromere/kinetochore assembly. Cell Rep.

[53] Lawrence KS, Chau T, Engebrecht J (2015). DNA damage response and spindle assembly checkpoint function throughout the cell cycle to ensure genomic integrity. PLoS Genet.

[54] Eferl R, Wagner EF (2003). AP-1: a double-edged sword in tumorigenesis. Nat Rev Cancer.

[55] Angel P, Hattori K, Smeal T, Karin M (1988). The jun proto-oncogene is positively autoregulated by its product, Jun/AP-1. Cell.

[56] Foletta VC, Segal DH, Cohen DR (1998). Transcriptional regulation in the immune system: all roads lead to AP-1. J Leukoc Biol.

[57] Mruthyunjaya S, Rumma M, Ravibhushan G, Anjali S, Padma S (2011). c-Jun/AP-1 transcription factor regulates laminin-1-induced neurite outgrowth in human bone marrow mesenchymal stem cells: role of multiple signaling pathways. FEBS Lett.

[58] Christmann M, Tomicic MT, Aasland D, Berdelle N, Kaina B (2010). Three prime exonuclease I (TREX1) is Fos/AP-1 regulated by genotoxic stress and protects against ultraviolet light and benzo(a)pyrene-induced DNA damage. Nucleic Acids Res.

[59] McNulty SE, Barrett RM, Vogel-Ciernia A, Malvaez M, Hernandez N, Davatolhagh MF (2012). Differential roles for Nr4a1 and Nr4a2 in object location vs. object recognition long-term memory. Learn Mem.

[60] Kim JI, Jeon SG, Kim KA, Kim YJ, Song EJ, Choi J (2016). The pharmacological stimulation of Nurr1 improves cognitive functions via enhancement of adult hippocampal neurogenesis. Stem Cell Res.

[61] Yin K, Chhabra Y, Tropee R, Lim YC, Fane M, Dray E (2017). NR4A2 promotes DNA double-strand break repair upon exposure to UVR. Mol Cancer Res.

[62] Barbosa AC, Kim MS, Ertunc M, Adachi M, Nelson ED, McAnally J (2008). MEF2C, a transcription factor that facilitates learning and memory by negative regulation of synapse numbers and function. Proc Natl Acad Sci USA.

[63] Gurden H, Schiffmann SN, Lemaire M, Bohme GA, Parmentier M, Schurmans S (1998). Calretinin expression as a critical component in the control of dentate gyrus long-term potentiation induction in mice. Eur J Neurosci.

[64] Mitchell AC, Javidfar B, Pothula V, Ibi D, Shen EY, Peter CJ (2018). MEF2C transcription factor is associated with the genetic and epigenetic risk architecture of schizophrenia and improves cognition in mice. Mol Psychiatry.

[65] Walton NM, Zhou Y, Kogan JH, Shin R, Webster M, Gross AK (2012). Detection of an immature dentate gyrus feature in human schizophrenia/bipolar patients. Transl Psychiatry.

[66] Yamada K, Gerber DJ, Iwayama Y, Ohnishi T, Ohba H, Toyota T (2007). Genetic analysis of the calcineurin pathway identifies members of the EGR gene family, specifically EGR3, as potential susceptibility candidates in schizophrenia. Proc Natl Acad Sci USA.

[67] Kim SH, Song JY, Joo EJ, Lee KY, Ahn YM, Kim YS (2010). EGR3 as a potential susceptibility gene for schizophrenia in Korea. Am J Med Genet Part B, Neuropsychiatr Genet Off Publ Int Soc Psychiatr Genet.

[68] Zhang R, Lu S, Meng L, Min Z, Tian J, Valenzuela RK (2012). Genetic evidence for the association between the early growth response 3 (EGR3) gene and schizophrenia. PloS One.

[69] Mexal S, Frank M, Berger R, Adams CE, Ross RG, Freedman R (2005). Differential modulation of gene expression in the NMDA postsynaptic density of schizophrenic and control smokers. Brain Res Mol Brain Res.

[70] Etemadikhah M, Niazi A, Wetterberg L, Feuk L (2020). Transcriptome analysis of fibroblasts from schizophrenia patients reveals differential expression of schizophrenia-related genes. Sci Rep.

[71] Leal G, Bramham CR, Duarte CB (2017). BDNF and hippocampal synaptic plasticity. Vitam Horm.

[72] Brown JR, Ye H, Bronson RT, Dikkes P, Greenberg ME (1996). A defect in nurturing in mice lacking the immediate early gene fosB. Cell.

[73] Hiroi N, Brown JR, Haile CN, Ye H, Greenberg ME, Nestler EJ (1997). FosB mutant mice: loss of chronic cocaine induction of Fos-related proteins and heightened sensitivity to cocaine’s psychomotor and rewarding effects. Proc Natl Acad Sci USA.

[74] McKinnon PJ (2009). DNA repair deficiency and neurological disease. Nat Rev Neurosci.

[75] McKinnon PJ (2013). Maintaining genome stability in the nervous system. Nat Neurosci.

[76] Suberbielle E, Sanchez PE, Kravitz AV, Wang X, Ho K, Eilertson K (2013). Physiologic brain activity causes DNA double-strand breaks in neurons, with exacerbation by amyloid-beta. Nat Neurosci.

[77] Madabhushi R, Gao F, Pfenning AR, Pan L, Yamakawa S, Seo J (2015). Activity-induced DNA breaks govern the expression of neuronal early-response genes. Cell.

[78] Li L, Yun SH, Keblesh J, Trommer BL, Xiong H, Radulovic J (2007). Egr3, a synaptic activity regulated transcription factor that is essential for learning and memory. Mol Cell Neurosci.

[79] Shin SH, Kim I, Lee JE, Lee M, Park JW (2020). Loss of EGR3 is an independent risk factor for metastatic progression in prostate cancer. Oncogene.

[80] Andley UP, Patel HC, Xi JH, Bai F (2004). Identification of genes responsive to UV-A radiation in human lens epithelial cells using complementary DNA microarrays. Photochem Photobio.

[81] Catts VS, Catts SV, Jablensky A, Chandler D, Weickert CS, Lavin MF (2012). Evidence of aberrant DNA damage response signalling but normal rates of DNA repair in dividing lymphoblasts from patients with schizophrenia. World J Biol Psychiatry.

[82] Madabhushi R, Pan L, Tsai LH (2014). DNA damage and its links to neurodegeneration. Neuron.

[83] Wang M, Wei PC, Lim CK, Gallina IS, Marshall S, Marchetto MC (2020). Increased neural progenitor proliferation in a hiPSC model of autism induces replication stress-associated genome instability. Cell Stem Cell.

[84] Markkanen E, Meyer U, Dianov GL (2016). DNA damage and repair in schizophrenia and autism: implications for cancer comorbidity and beyond. Int J Mol Sci.

[85] Ceylan D, Yilmaz S, Tuna G, Kant M, Er A, Ildiz A (2020). Alterations in levels of 8-Oxo-2’-deoxyguanosine and 8-Oxoguanine DNA glycosylase 1 during a current episode and after remission in unipolar and bipolar depression. Psychoneuroendocrinology.

[86] Schurmans S, Schiffmann SN, Gurden H, Lemaire M, Lipp HP, Schwam V (1997). Impaired long-term potentiation induction in dentate gyrus of calretinin-deficient mice. Proc Natl Acad Sci USA.

[87] Lyons GE, Micales BK, Schwarz J, Martin JF, Olson EN (1995). Expression of mef2 genes in the mouse central nervous system suggests a role in neuronal maturation. J Neurosci Off J Soc Neurosci.

[88] Al-Jaberi N, Lindsay S, Sarma S, Bayatti N, Clowry GJ (2015). The early fetal development of human neocortical GABAergic interneurons. Cereb Cortex.

[89] Eagle AL, Gajewski PA, Yang M, Kechner ME, Al Masraf BS, Kennedy PJ (2015). Experience-dependent induction of hippocampal DeltaFosB controls learning. J Neurosci.

[90] Kwon W, Kim HS, Jeong J, Sung Y, Choi M, Park S (2018). Tet1 overexpression leads to anxiety-like behavior and enhanced fear memories via the activation of calcium-dependent cascade through Egr1 expression in mice. FASEB J.

[91] O’Donovan KJ, Wilkens EP, Baraban JM (1998). Sequential expression of Egr-1 and Egr-3 in hippocampal granule cells following electroconvulsive stimulation. J Neurochem.

