# Supplementary material for: Identification of activity-induced Egr3-dependent genes reveals genes associated with DNA damage response and schizophrenia
Source: Transl Psychiatry. 2022 Aug 8;12:320. doi: 10.1038/s41398-022-02069-8 (PMC9360026; doi:10.1038/s41398-022-02069-8)
Supplement: Supplementary file 1 — Supplemental Figure 1 [file 41398_2022_2069_MOESM1_ESM.pdf]

Figure S1.

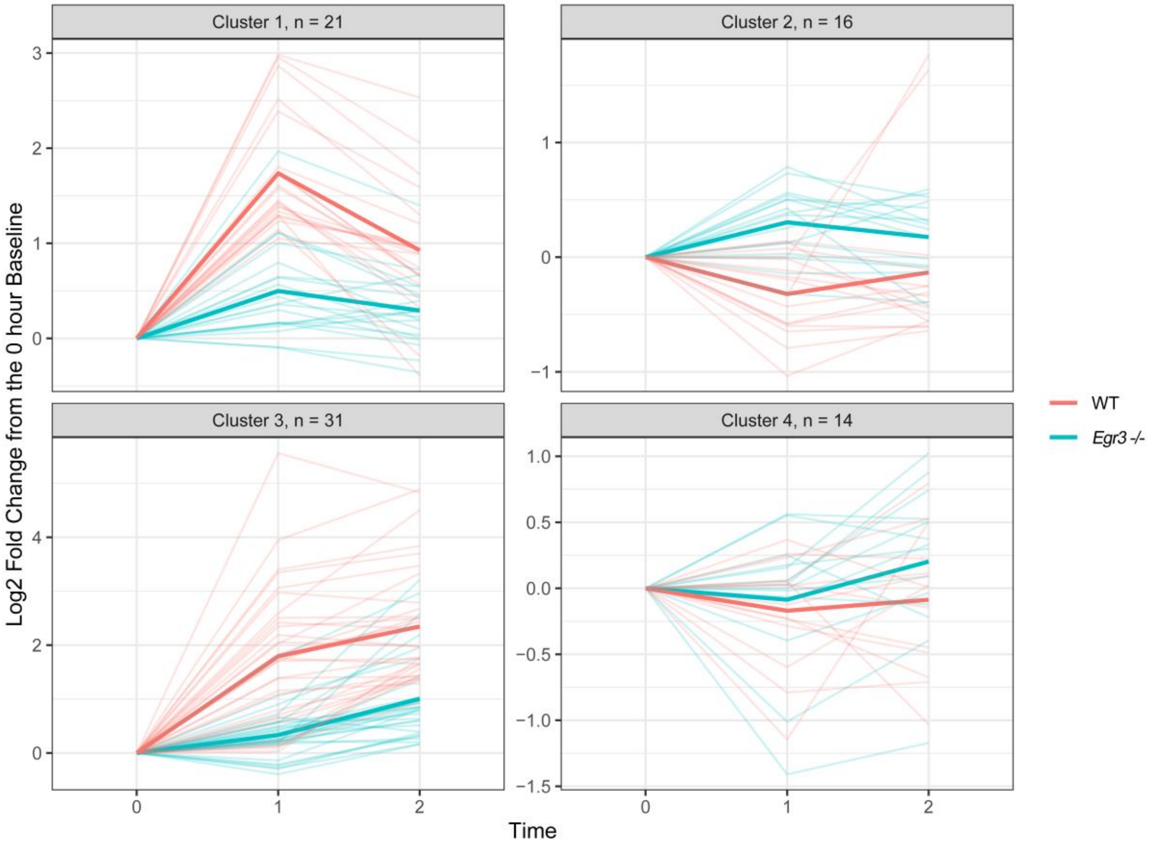

Figure S1. Differentially expressed genes fall into four clusters.
The four clusters of DEGs generated by the k-mean clustering analysis are visualized here to represent the pattern of gene expression changes across the different timepoints relative to the baseline (timepoint = 0). The number of clusters (k) was determined using the “elbow” method. The n in the headings of each panel represents the number of genes in each cluster. The transparent lines show the log2 fold change relative to the baseline for each gene, and the heavy lines show the average of these patterns per group. The colors denote the two groups (WT and Egr3-/-).
