# Supplementary material for: Identification of activity-induced Egr3-dependent genes reveals genes associated with DNA damage response and schizophrenia
Source: Transl Psychiatry. 2022 Aug 8;12:320. doi: 10.1038/s41398-022-02069-8 (PMC9360026; doi:10.1038/s41398-022-02069-8)
Supplement: Supplementary file 2 — Supplemental Figure 2 [file 41398_2022_2069_MOESM2_ESM.pdf]

Figure S2.

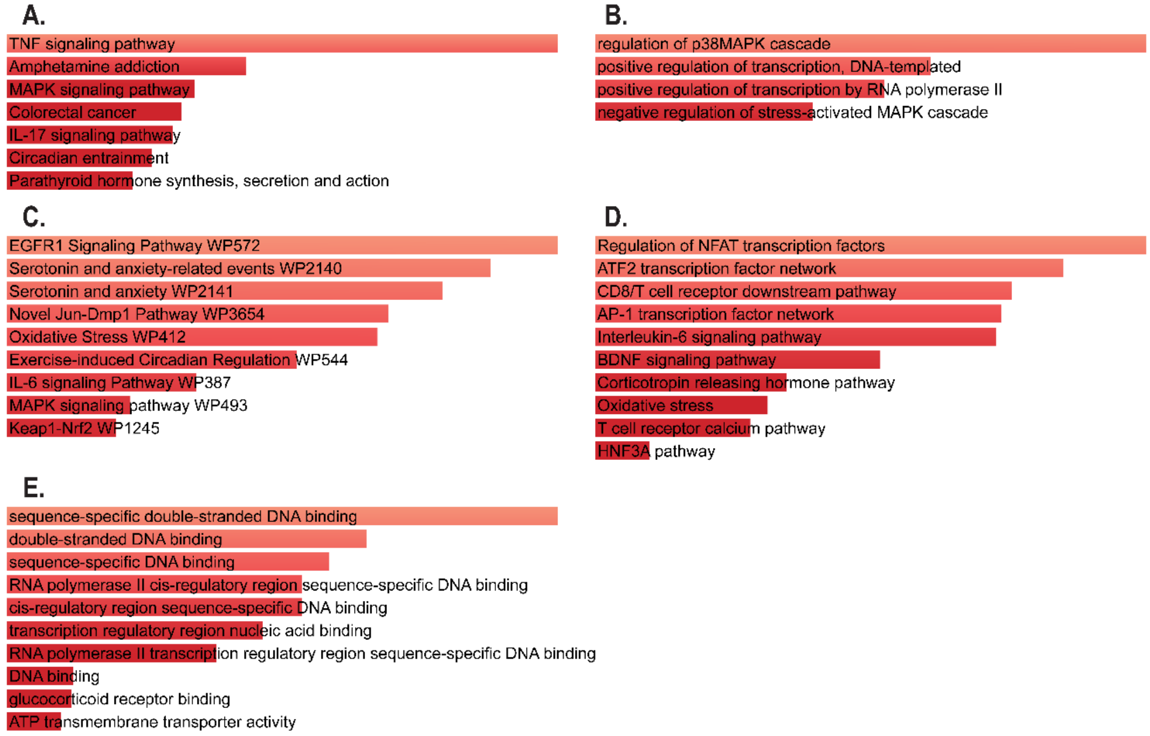

Figure S2. Enrichr Pathway Results for Cluster 1 DEGs.
Gene set enrichment analyses were performed on the list of DEGs for each cluster using the Enrichr web tool. Results of cluster 1 DEGs show the most significantly enriched biological processes represented in this cluster. The length of the bar and the brightness of the color represents the significance of each specific gene-set or pathway term. The top ten pathways are presented in order from most significant (brightest color) to least significant (darkest color) adjusted p-value. Only pathways that reached significance (adjusted p value < 0.05) are shown. Each of the five gene set libraries analyzed returned statistically significant pathways in the cluster 1 analyses. These are: **A.** KEGG 2019 Mouse, **B.** Gene Ontology (GO) Biological Process 2021, **C.** WikiPathways 2019 Mouse, **D.** BioPlanet 2019, and **E.** GO Molecular Function 2021.
