# Supplementary material for: Identification of activity-induced Egr3-dependent genes reveals genes associated with DNA damage response and schizophrenia
Source: Transl Psychiatry. 2022 Aug 8;12:320. doi: 10.1038/s41398-022-02069-8 (PMC9360026; doi:10.1038/s41398-022-02069-8)
Supplement: Supplementary file 3 — Supplemental Figure 3 [file 41398_2022_2069_MOESM3_ESM.pdf]

Figure S3.

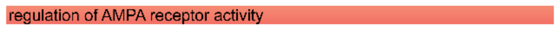

Figure S3. Enrichr Pathway Results for Cluster 2 DEGs.
Enrichr pathway analysis results for cluster 2 DEGs revealed only one statistically significant pathway. This was from the gene set library Gene Ontology (GO) Biological Process 2021.
