# Supplementary material for: Identification of activity-induced Egr3-dependent genes reveals genes associated with DNA damage response and schizophrenia
Source: Transl Psychiatry. 2022 Aug 8;12:320. doi: 10.1038/s41398-022-02069-8 (PMC9360026; doi:10.1038/s41398-022-02069-8)
Supplement: Supplementary file 4 — Supplemental Figure 4 [file 41398_2022_2069_MOESM4_ESM.pdf]

Figure S4.

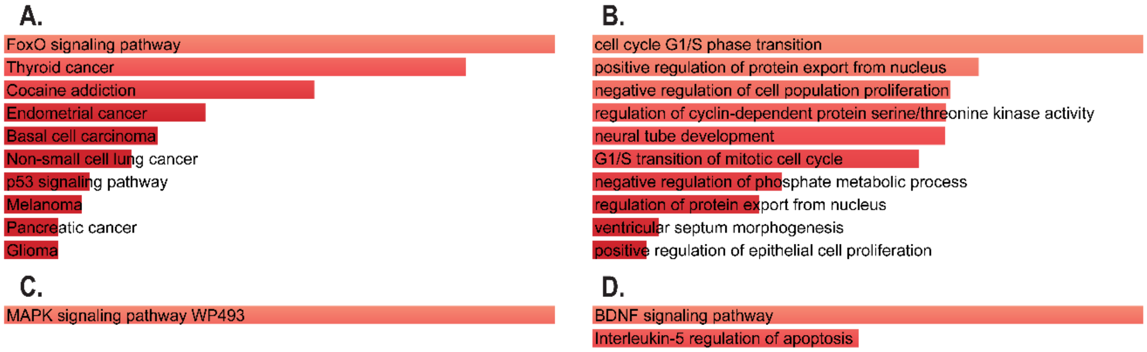

Figure S4. Enrichr Pathway Results for Cluster 3 DEGs.
Enrichr pathway analysis results of cluster 3 DEGs show the most significantly enriched biological processes represented in this cluster. The length of the bar and the brightness of the color represents the significance of each specific gene-set or pathway term. The top ten pathways are presented in order from most significant (brightest color) to least significant (darkest color) adjusted p-value. Only pathways that reached significance (adjusted p value < 0.05) are shown. The gene set libraries that returned statistically significant pathway terms in the cluster 3 analyses are as follows: **A.** KEGG 2019 Mouse, **B.** Gene Ontology (GO) Biological Process 2021, **C.** WikiPathways 2019 Mouse, and **D.** BioPlanet 2019. Enrichr pathway analyses of cluster 4 DEG revealed no statistically significant (adjusted p-value < 0.05) results among the five gene set enrichment libraries queried.
