# Supplementary material for: Identification of activity-induced Egr3-dependent genes reveals genes associated with DNA damage response and schizophrenia
Source: Transl Psychiatry. 2022 Aug 8;12:320. doi: 10.1038/s41398-022-02069-8 (PMC9360026; doi:10.1038/s41398-022-02069-8)
Supplement: Supplementary file 5 — Supplemental Figure 5 [file 41398_2022_2069_MOESM5_ESM.pdf]

Figure S5.

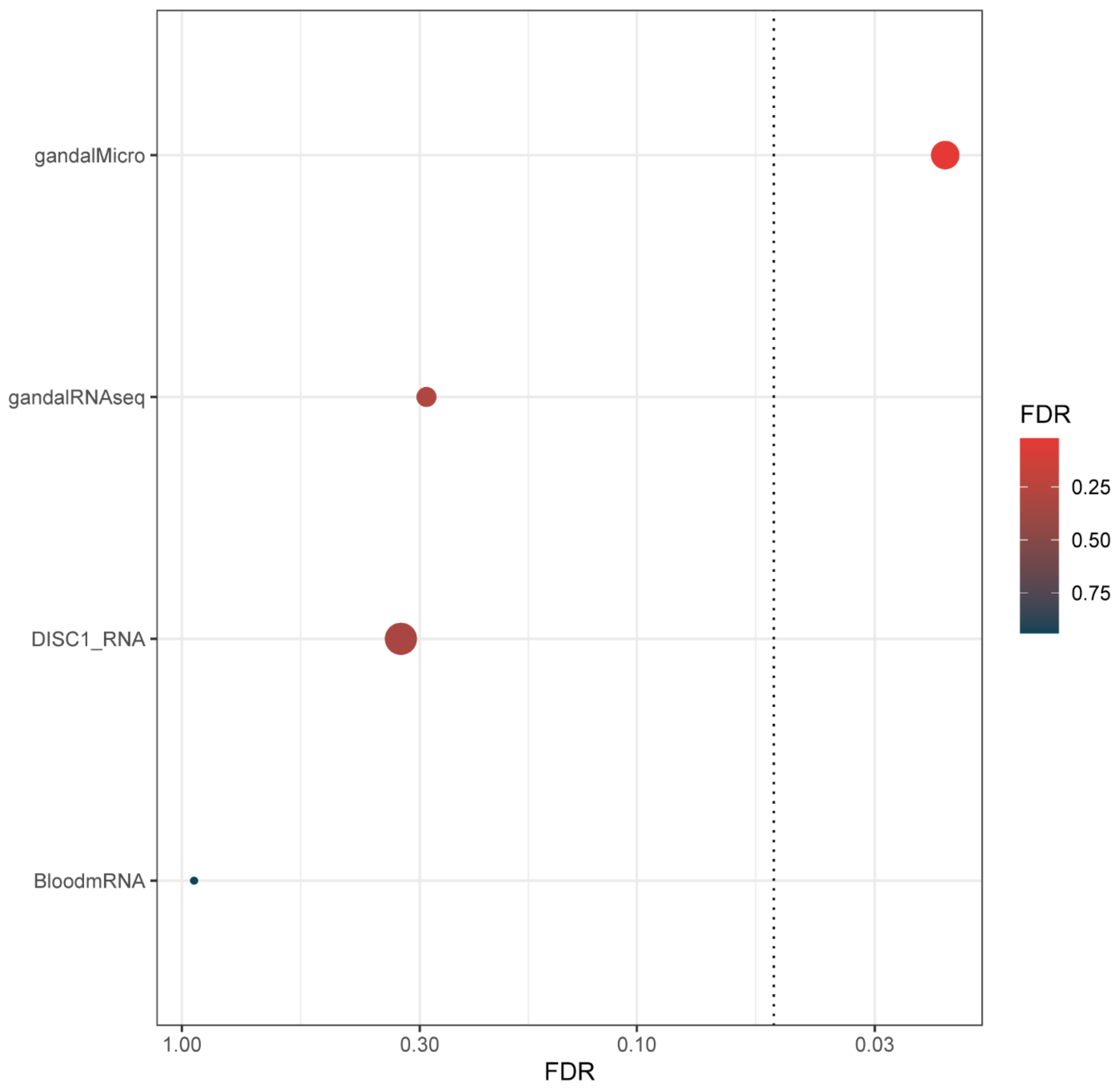

Figure S5. Gene set enrichment analysis against schizophrenia transcriptional signatures. Gene set enrichment analysis using a hypergeometric test to analyze the overlap between the DEGs in WT vs *Egr3*-/- mice 1 hour following ECS and the DEGs in schizophrenia transcriptional datasets.
