# Supplementary material for: Identification of activity-induced Egr3-dependent genes reveals genes associated with DNA damage response and schizophrenia
Source: Transl Psychiatry. 2022 Aug 8;12:320. doi: 10.1038/s41398-022-02069-8 (PMC9360026; doi:10.1038/s41398-022-02069-8)
Supplement: Supplementary file 6 — Supplemental Figure 6 [file 41398_2022_2069_MOESM6_ESM.pdf]

Figure S6.

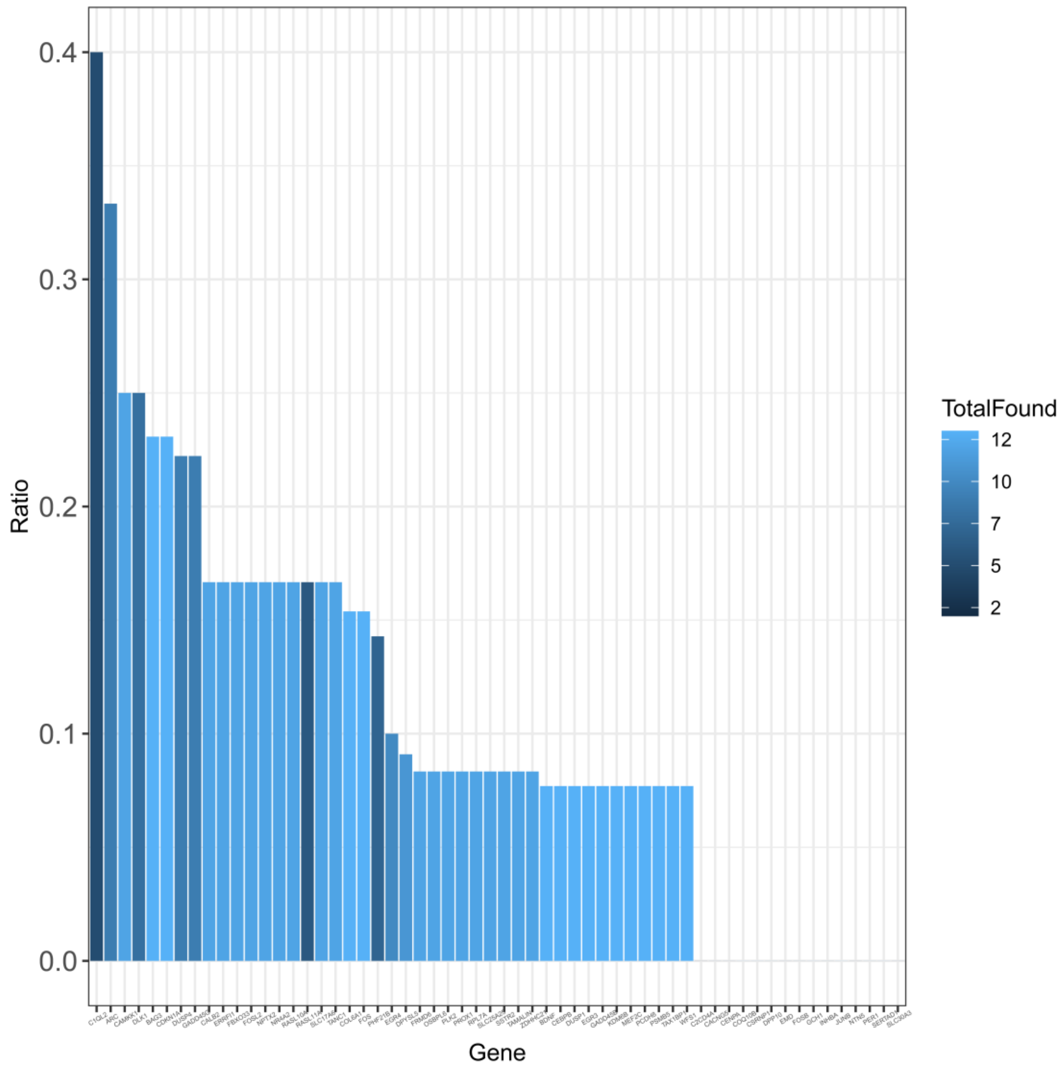

Figure S6. Proportion of schizophrenia studies in which each DEG is identified. Thirteen published gene expression studies in schizophrenia were queried for the 58 DEGs in Egr3-/- compared with WT mouse hippocampus 1 hr. following ECS for which human homologues were present in at least one of the human schizophrenia studies. The histogram plot showing the proportion of the 13 studies in which each gene was found to be differentially expressed in schizophrenia patient samples compared with controls (fold change is at least 15% and p-value ≤ 0.05).
